# Exploring the larval transcriptome of the common sole (*Solea solea* L.)

**DOI:** 10.1186/1471-2164-14-315

**Published:** 2013-05-10

**Authors:** Serena Ferraresso, Alessio Bonaldo, Luca Parma, Stefano Cinotti, Paola Massi, Luca Bargelloni, Pier Paolo Gatta

**Affiliations:** 1 Department of Comparative Biomedicine and Food Science, University of Padova, Viale dell’Università 16, Legnaro, PD 35020, Italy; 2 Department of Veterinary Medical Sciences, Alma Mater Studiorum, University of Bologna, via Tolara di Sopra 50, Ozzano Emilia, BO, 40064, Italy; 3 Istituto Zooprofilattico Sperimentale della Lombardia e dell’Emilia, via Bianchi 9, Brescia, 24125, Italy; 4 Istituto Zooprofilattico Sperimentale della Lombardia e dell’Emilia, Sezione di Forlì, Via Marchini 1, Forlì, 47122, Italy

**Keywords:** Solea solea, Flatfish, Larval development, Metamorphosis, Transcriptome, Gene expression

## Abstract

**Background:**

The common sole (*Solea solea*) is a promising candidate for European aquaculture; however, the limited knowledge of the physiological mechanisms underlying larval development in this species has hampered the establishment of successful flatfish aquaculture. Although the fact that genomic tools and resources are available for some flatfish species, common sole genomics remains a mostly unexplored field. Here, we report, for the first time, the sequencing and characterisation of the transcriptome of *S. solea* and its application for the study of molecular mechanisms underlying physiological and morphological changes during larval-to-juvenile transition.

**Results:**

The *S. solea* transcriptome was generated from whole larvae and adult tissues using the Roche 454 platform. The assembly process produced a set of 22,223 Isotigs with an average size of 726 nt, 29 contigs and a total of 203,692 singletons. Of the assembled sequences, 75.2% were annotated with at least one known transcript/protein; these transcripts were then used to develop a custom oligo-DNA microarray. A total of 14,674 oligonucleotide probes (60 nt), representing 12,836 transcripts, were *in situ* synthesised onto the array using Agilent non-contact ink-jet technology. The microarray platform was used to investigate the gene expression profiles of sole larvae from hatching to the juvenile form. Genes involved in the ontogenesis of the visual system are up-regulated during the early stages of larval development, while muscle development and anaerobic energy pathways increase in expression over time. The gene expression profiles of key transcripts of the thyroid hormones (TH) cascade and the temporal regulation of the GH/IGF1 (growth hormone/insulin-like growth factor I) system suggest a pivotal role of these pathways in fish growth and initiation of metamorphosis. Pre-metamorphic larvae display a distinctive transcriptomic landscape compared to previous and later stages. Our findings highlighted the up-regulation of gene pathways involved in the development of the gastrointestinal system as well as biological processes related to folic acid and retinol metabolism. Additional evidence led to the formation of the hypothesis that molecular mechanisms of cell motility and ECM adhesion may play a role in tissue rearrangement during common sole metamorphosis.

**Conclusions:**

Next-generation sequencing provided a good representation of the sole transcriptome, and the combination of different approaches led to the annotation of a high number of transcripts. The construction of a microarray platform for the characterisation of the larval sole transcriptome permitted the definition of the main processes involved in organogenesis and larval growth.

## Background

Flatfish (order Pleuronectiformes) include 716 different species worldwide, mostly marine, which undergo a unique developmental process during the larval-to-juvenile transition in which one eye migrates across the top of the skull to lie adjacent to the other eye on the opposite side, while the body flattens and lies on the eyeless side [1]. Members of the order Pleuronectiformes also represent an important food resource as low-fat fish with a white, flavourful flesh that is highly acceptable to consumers. Despite their economic importance, flatfish production is still much lower than that of salmonids, cyprinids or other marine species such as the European sea bass and the gilthead sea bream. In Europe, the main cultured flatfish species are turbot, Atlantic halibut, and, to a lesser extent, the Senegalese sole and the common sole [2]. The limited knowledge of the basic biology of flatfish has hampered the development of efficient aquaculture practices for these species. The highest mortalities during the entire fish life cycle occur during larval development, particularly during the transition from endogenous to exogenous feeding, weaning and metamorphosis [3,4]. Flatfish metamorphosis and other developmental events involve drastic morphological and physiological changes, the molecular basis of which remains poorly understood. The transition from larval to juvenile stage involves the development of most organs and tissues, the maturation of different physiological functions and the establishment of the immune system; therefore, this transition represents a critical step in flatfish farming. In fact, the current bottlenecks in flatfish production are mainly associated with the optimisation of larval culture and nutrition as well as the high larval mortality due to infectious diseases. The limited knowledge of the physiological mechanisms underlying larval development has hampered the establishment of a successful flatfish aquaculture [5,6]. In recent years, functional genomics and proteomics approaches have been applied to flatfish research in order to enhance the knowledge of the biology of these species and shed light on the molecular mechanisms underlying different physiological processes [7-12]. The identification and characterisation of genes and gene networks controlling traits of commercial interest such as growth rate, reproduction and disease resistance would facilitate the optimisation of production and management procedures in the industry.

The common sole (*Solea solea*), which is characterised by high flesh quality and high market value, is a very promising candidate for European aquaculture. The development of a robust sole aquaculture production will also help reduce fishing pressure on wild sole populations, which are currently overexploited. As for other flatfish species, however, several critical bottlenecks must be addressed in order to establish large scale sole farming production. Feeding behaviour, susceptibility to diseases, stocking density as well as juvenile mortality represent key critical factors for sole aquaculture. Although genomic tools and resources are available for some flatfish species (*e.g.* turbot, Atlantic halibut, Senegalese sole), common sole genomics remains a mostly unexplored area of research.

Here, we report for the first time the sequencing and characterisation of the transcriptome of *S. solea,* focusing on larval and juvenile stages. After transcriptome sequencing and annotation, an oligo-DNA microarray for the detection of 12,836 unique transcripts was developed and applied to the study of molecular mechanisms underlying physiological and morphological changes during the larval-to-juvenile transition.

## Results

### S. solea larval transcriptome assembly and annotation

High-throughput sequencing of a *S. solea* cDNA library generated a total of 909,466 sequences (882,214 after trimming), with a mean length of 245 nucleotides (nt). Newly produced sequences were assembled together with already available mRNA sequences (314,486; see Methods) with Newbler 2.6. The software produced a set of 22,223 Isotigs (grouped into 20,281 Isogroups) with an average size of 726 nt (N50 Isotig Size 808 nt), 29 contigs and a total of 203,692 singletons. The final number of aligned reads was 941,883 (78.71%) (number assembled = 852,258). All Isotigs and contigs have been stored in the public database Transcriptome Shotgun Assembly Sequence Database (TSA, [13]) under accession number GAAQ00000000; transcripts sequences can be retrieved by using the sequence name as the search criteria. The putative identities of the assembled sequences were obtained by running Blastx and Blastn similarity searches on 18 different protein and nucleotide databases. Of 22,252 unique sequences, 16,731 (75.2%) showed at least one significant match with a known transcript or protein. All transcripts and corresponding annotations are listed in Additional file 1. After further clustering by proteome mapping, a total of 1,346 Isotigs (1,196 showing the same annotation with all 5 fish species) were filtered out, yielding a total of 15,385 unique annotated transcripts, which were employed for microarray design. The Simple Sequence Repeats (SSRs) content of all Isotigs and contigs was also investigated. Of 22,252 sequences examined, 3,612 contained at least one SSR, with 638 sequences showing more than one SSR, for a total of 4,402 identified SSRs. The number of repeated dinucleotides was 2,622, with “AC” and “TG” SSRs being the most frequent (520 SSRs and 506 SSRs, respectively). The number of repeated trinucleotides was 1,486 (the “TTC” trinucleotide was the most frequent, with 89 SSRs). The number of tetranucleotide repeats was 247, while penta- and hexanucleotide microsatellites accounted for 34 and 16 SSRs, respectively.

### Global gene expression analysis

**Figure 1:**
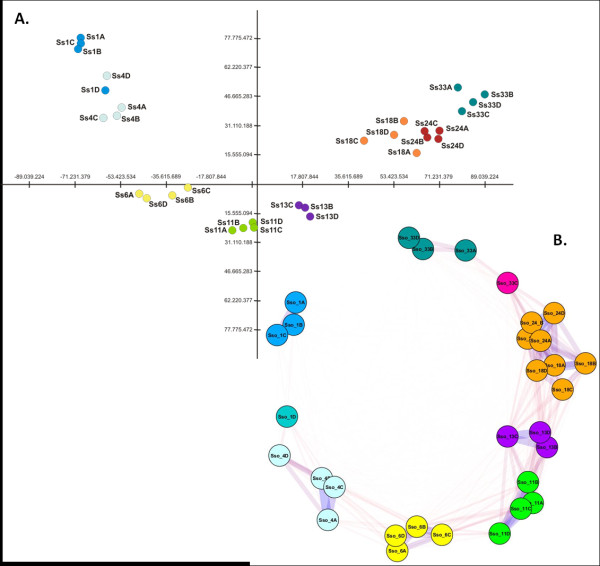
**Global analyses of larval gene expression profiles. A. **Principal Component Analysis (PCA) on the entire probe set. **B. **Sample clustering through AutoSOME. Ss: Solea solea, larval stages are indicated by number, biological replicates are distinguished by letters A, B, C and D.

Raw and normalised fluorescence data from all microarray experiments have been deposited in the GEO database [14] under accession number GSE41261. Three different clustering methods were employed in order to group samples according to their gene expression profiles. Principal Component Analysis (PCA) on the entire probe set divided 31 sole pool samples into 8 separate groups (Figure 1A), with the first and second components explaining nearly 2/3 (62.2%) of the variation in the entire data set. The main separation into groups of samples along the first axis closely reflects (with the sole exception of Ss_1D) the temporal component of larval development. However, a “horseshoe effect” with a curved distortion along the Y axis was clearly visible. Sample separation along the second axis sets 11 and 13 dph larvae at the opposite end compared to the position of stage 1 (1 dph) and 33 (33 dph).

The same dataset was analysed using a SOM-based clustering method, AutoSOME, which placed all samples into 7 major clusters (Figure 1B), with Sso_1D and Sso_33C highlighted as singletons. As in the PCA analysis, the sample classification reflects the temporal scale of developmental stages. Pairwise affinities between samples (the fraction of times two samples are co-clustered), however, revealed a stronger relationship between 11 dph and 13 dph larvae as well as 18 dph and 24 dph individuals. The latter two stages were grouped in the same cluster. Comparable results were obtained with unsupervised hierarchical clustering (HCL) analysis (data not shown).

### Transcriptional changes over time

Quantitative correlation analysis as implemented in the software Significant Analysis of Microarray (SAM) [15] was used in order to identify genes whose expression either increased or decreased over time. A total of 2,209 probes were positively correlated and 4,376 transcripts were negatively correlated with time of larval development. The functional annotation of significant genes using DAVID pinpointed a few pathways of particular interest (see Table 1). Among up-regulated genes, the most significant KEGG pathways are related to muscle development/contraction and glucose metabolism, while the Hedgehog signalling pathway (dre04340) and Wnt signalling pathway (dre04310) are among the most significant pathways represented by genes down-regulated over time. Key components of muscle development and function such as *caveolin 3* (N_isotig07042), *troponin T* (N_isotig13004), *tropomyosin* (P_isotig00564) and *cholinergic receptor, nicotinic, alpha 1* (CHNRA*,* N_isotig07602), which modulate muscle contraction as well as several form of *myosin*, display an increase in gene expression over time (Table 1, an heatmat showing gene expression values is reported in Additional file 2). Glucose metabolism, particularly glycolysis, is represented by several genes displaying the same trend; *aldolase a* (P_contig00403*), glucose phosphate isomerase b* (N_isotig03674), g*lyceraldehyde-3-phosphate dehydrogenase* (GAPDH, N_isotig18841), *lactate dehydrogenase* (P_isotig00860) and many others increase in expression more than 20-fold from 1 to 33 dph.

**Table 1:** Genes up- or down-regulated over time

**MUSCLE DEVELOPMENT**		
**Probe Name**	**Ensembl Acc. Number**	**Gene name**
**N_isotig14746**	ENSDARG00000032976	*Cardiac myosin light chain-1*
**P_isotig00564**	ENSDARG00000023963	*Tropomyosin*
**N_isotig13004**	ENSDARG00000020610	*Troponin T*
**N_isotig07042**	ENSDARG00000024141	*Caveolin 3*
**N_isotig07602**	ENSDARG00000009021	*Cholinergic receptor, nicotinic, alpha 1 (CHNRA)*
**N_isotig21306**	ENSDARG00000071433	*Slow myosin heavy chain 2*
**N_isotig01223**	ENSDARG00000045242	*Slow myosin heavy chain 3*
**N_isotig04075**	ENSDARG00000028213	*Titin a*
**N_isotig11618**	ENSDARG00000000563	*Titin b*
**N_isotig08672**	ENSDARG00000019342	*Cholinergic receptor, nicotinic, delta polypeptide*
**N_isotig19778**	ENSDARG00000031756	*Myocyte enhancer factor 2a*
**N_isotig05817**	ENSDARG00000054942	*Lectin, galactoside-binding, soluble, 1 (galectin 1)-like 1*
**P_isotig17511**	ENSDARG00000026473	*Sine oculis homeobox homolog 1b*
**N_isotig01855**	ENSDARG00000006112	*Ras-related C3 botulinum toxin substrate 1*
**N_isotig03727**	ENSDARG00000034240	*Capping protein muscle Z-line, alpha 1*
**P_isotig01165**	ENSDARG00000046004	*Capping protein muscle Z-line, beta*
**P_isotig18441**	ENSDARG00000023797	*Ryanodine receptor 1b*
**N_isotig17357**	ENSDARG00000019096	*Myosin, light polypeptide 7*
**GLUCOSE METABOLISM**		
**Probe Name**	**Ensembl Acc. Number**	**Gene name**
**N_contig01740**	ENSDARG00000003191	*Pyruvate kinase, muscle, b*
**N_isotig13675**	ENSDARG00000004059	*Galactokinase*
**N_isotig03674**	ENSDARG00000005161	*Glucose phosphate isomerase b*
**P_isotig13251**	ENSDARG00000005423	*Phosphoglycerate mutase 1a*
**P_contig00403**	ENSDARG00000011665	*Aldolase a, fructose-bisphosphate, a*
**N_isotig03547**	ENSDARG00000014179	*Phosphofructokinase, muscle a*
**P_isotig10593**	ENSDARG00000016875	*Glycogen synthase 1*
**P_isotig18039**	ENSDARG00000019702	*Aldolase c, fructose-bisphosphate*
**N_isotig03683**	ENSDARG00000022456	*Enolase 1, (alpha)*
**N_isotig04393**	ENSDARG00000026964	*Hexokinase 2*
**N_isotig06502**	ENSDARG00000028088	*Galactokinase 1*
**N_isotig03558**	ENSDARG00000030604	*Phosphorylase kinase, gamma 1 (PHKG1a)*
**N_isotig18841**	ENSDARG00000039914	*Glyceraldehyde-3-phosphate dehydrogenase*
**P_isotig00860**	ENSDARG00000040856	*Lactate dehydrogenase A4*
**P_isotig00400**	ENSDARG00000043180	*Glycerol-3-phosphate dehydrogenase 1b*
**N_contig00167**	ENSDARG00000054191	*Phosphoglycerate kinase 1*
**N_isotig05258**	ENSDARG00000057571	*Phosphoglycerate mutase 2 (muscle)*
**N_isotig12521**	ENSDARG00000057630	*Aldose 1-epimerase*
**N_isotig06792**	ENSDARG00000060797	*Phosphofructokinase, muscle b*
**P_isotig13923**	ENSDARG00000062998	*Peptidoglycan recognition protein 2*
**P_isotig03551**	ENSDARG00000070826	*2,3-bisphosphoglycerate mutase*
**N_isotig20169**	ENSDARG00000071076	*Similar to L-lactate dehydrogenase B chain*
**HEDGEHOG SIGNALLING PATHWAY**		
**Probe Name**	**Ensembl Acc. Number**	**Gene name**
**N_isotig03639**	ENSDARG00000052131	*GLI-Kruppel family member GLI3*
**N_isotig05619**	ENSDARG00000008370	*Casein kinase 1, delta a*
**N_isotig07032**	ENSDARG00000005458	*Casein kinase 1, gamma 2a*
**N_isotig10399**	ENSDARG00000017803	*Glycogen synthase kinase 3 beta (GSK3B)*
**N_isotig10687**	ENSDARG00000034056	*Casein kinase 1, gamma 2b*
**N_isotig12392**	ENSDARG00000004965	*Bone morphogenetic protein 5*
**N_isotig15047**	ENSDARG00000071107	*Wingless-type MMTV integration site family,7Bb (WNT7)*
**N_isotig19239**	ENSDARG00000060397	*Hedgehog interacting protein (HiP)*
**N_isotig20428**	ENSDARG00000017230	*F-box and WD-40 domain protein 11b (FBXW11)*
**N_isotig21462**	ENSDARG00000014134	*Similar to cAMP-dependent protein kinase (PKA C-alpha)*
**P_isotig04440**	ENSDARG00000059125	*Protein kinase, cAMP-dependent, catalytic, beta*
**P_isotig07996**	ENSDARG00000060649	*Megalin, low density lipoprotein-related protein 2 (LRP2)*
**P_isotig12515**	ENSDARG00000052674	*Casein kinase 1, alpha 1*
**P_isotig16544**	ENSDARG00000015554	*Zic family member 2*
**P_isotig17732**	ENSDARG00000063230	*Bone morphogenetic protein 7b*
**P_isotig18139**	ENSDARG00000068567	*Sonic hedgehog-like; Sonic hedgehog a*
**WNT SIGNALLING PATHWAY**		
**Probe Name**	**Ensembl Acc. Number**	**Gene name**
**P_isotig22061**	ENSDARG00000004305	*Vang-like 1 (van gogh, Drosophila)*
**P_isotig10261**	ENSDARG00000007791	*Protein phosphatase 2 (formerly 2A), regulatory subunit, beta*
**P_isotig04271**	ENSDARG00000009689	*Dishevelled associated activator of morphogenesis 1*
**P_isotig18727**	ENSDARG00000009870	*Mitogen-activated protein kinase 8*
**N_isotig02405**	ENSDARG00000013582	*Similar to Casein kinase II subunit alpha (CK II)*
**N_isotig21462**	ENSDARG00000014134	*Similar to cAMP-dependent protein kinase (PKA C-alpha)*
**N_isotig04292**	ENSDARG00000014571	*Catenin, beta 2*
**N_isotig02225**	ENSDARG00000014731	*Calcyclin binding protein*
**N_isotig20428**	ENSDARG00000017230	*F-box and WD-40 domain protein 11b*
**N_isotig10399**	ENSDARG00000017803	*Glycogen synthase kinase 3 beta*
**P_isotig06163**	ENSDARG00000019239	*Cullin 1a*
**N_isotig12285**	ENSDARG00000025747	*Mitogen-activated protein kinase 10*
**P_isotig09852**	ENSDARG00000027397	*Vang-like 2 (van gogh, Drosophila)*
**AS_isotig13833**	ENSDARG00000031894	*Lymphocyte enhancer binding factor 1*
**P_isotig13436**	ENSDARG00000038954	*Beta-catenin-interacting protein*
**N_isotig20334**	ENSDARG00000039041	*Secreted frizzled-related protein 5*
**P_isotig04697**	ENSDARG00000044062	*C-terminal binding protein 2*
**N_isotig16673**	ENSDARG00000045444	*Frizzled homolog 8a*
**P_isotig12515**	ENSDARG00000052674	*Casein kinase 1, alpha 1*
**N_isotig03709**	ENSDARG00000053020	*Protein phosphatase 2 (formerly 2A), catalytic subunit A*
**P_isotig14067**	ENSDARG00000057007	*C-terminal binding protein 1*
**P_isotig10768**	ENSDARG00000060716	*Similar to Serine/threonine-protein kinase PRKX*
**N_isotig11316**	ENSDARG00000060976	*CREB binding protein b*
**N_isotig04935**	ENSDARG00000061308	*CREB binding protein a*
**N_isotig15047**	ENSDARG00000071107	*Wingless-type MMTV integration site family, 7Bb*
**P_isotig08176**	ENSDARG00000075226	*Smad4*
**P_isotig03701**	ENSDARG00000077776	*Casein kinase 2 beta*

By contrast, genes included in the pathways “Hedgehog signalling” and “Wnt signalling” displayed decreasing expression over time (see Table 1, Additional file 2 for corresponding heatmaps). These two key pathways are involved in developmental processes and control of asymmetric cell division. In particular, a large number of genes related to “Hedgehog signalling” displayed a decreasing temporal trend of expression, such as *sonic hedgehog-like* (P_isotig18139), *bone morphogenetic protein 7b* (P_isotig17732), *megalin* (P_isotig07996), and *hedgehog interacting protein* (N_isotig19239). Likewise, 28 genes belonging to “Wnt signalling”, which were members of the canonical pathway, the planar cell polarity (PCP) pathway, or the Wnt/Ca2+ pathway, were negatively correlated with time of larval development. Of particular interest are *neuropilin-1* and *transcription factor AP-2 alpha*, genes that control the development and differentiation of the neural crest.

### Transcriptional changes across larval stage transitions

A two-class unpaired SAM analysis was performed to identify transcriptional changes between two consecutive larval stages. The highest number of differentially expressed genes was found between 1 dph and 4 dph, with a total of 1,539 significant genes (974 over- and 565 under-expressed in 4 dph larvae), while only 120 genes (81 up- and 39 down-regulated) displayed a change in expression between 11 and 13 dph. To obtain a more comprehensive interpretation of the set of genes differentially expressed in each transition, enrichment analyses were performed using the software DAVID (see Methods). A complete list of Biological Process (BP) GO terms and KEGG pathways that were found to be significantly enriched is reported in Additional file 3.

#### Comparison of 1 and 4 dph larvae

**Figure 2:**
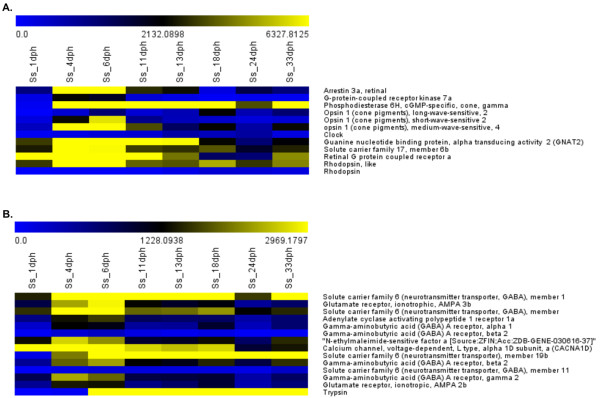
**Gene expression value over time of “Eye morphogenesis” and “Neurological system” pathways. **Heatmaps representing gene expression values in each developmental stage of genes involved in **A**. eye and **B**. neurological system morphogenesis.

A total of 29 GO-BP terms were found to be over-represented when 1 and 4 dph larvae were compared; the majority (17 out of 29) are related to the development of the neurological system and eye morphogenesis (*e.g.* GO:0050890 ~ cognition, GO:0007601 ~ visual perception, GO:0007602 ~ phototransduction and GO:0050877 ~ neurological system process). Strong over-expression of important photoreceptor components such as *opsin 1* (N_isotig12066, 44-fold increased), *arrestin 3* (N_isotig04447, 23-fold increased), *retinal G protein* (P_isotig10017, 3-fold increased) and *rhodopsin* (S_isotig03661, 7-fold increased) was identified in 4 dph larvae (see Figure 2A). A similar pattern can be observed also for key components of synapses and neurotransmitter release (see Figure 2B) such as *NSF* (N-ethylmaleimide-sensitive factor, N_isotig21343, 2-fold increased), *calcium channel, voltage-dependent, L type, alpha 1D subunit* (CACNA1D, N_isotig21406, 2-fold increased), and *solute carrier family 6, member 19* (P_isotig03793, 18-fold increased). Analysis of enriched KEGG terms confirmed these observations and identified *“Neuroactive ligand-receptor interaction”* (dre04080) as the most significant pathway (Additional file 3).

#### Comparison of 4 and 6 dph larvae

The functional annotation of genes that were differentially expressed between 4 dph and 6 dph larvae resulted in the identification of 13 BP terms in common with the previous larval transition as significantly enriched, all of which are related to visual perception and neurological system processes (see Figure 2), although the level of increase in expression was not identical to that in the previous comparison. Among genes up-regulated in 6 dph compared to 4 dph larvae, several GO terms are related to lipid metabolism (*i.e*. GO:0008610 ~ lipid biosynthetic process, GO:0006633 ~ fatty acid biosynthetic process and GO:0016125 ~ sterol metabolic process), including key genes such as *stearoyl-CoA desaturase* (N_isotig05992, 2.24 fold) and *ELOVL family member 5* (N_isotig05673, 3.76 fold), which display significant over-expression. This evidence is supported by KEGG analysis, which highlighted “Steroid biosynthesis” and “PPAR signalling pathway” as the most significantly enriched pathways.

#### Comparison of 6 and 11 dph larvae

The major evidence obtained when analyzing genes differentially expressed between 6 dph and 11 dph larvae is that all BP terms related to visual and neuronal processes remain enriched, although the corresponding genes display a significant down-regulation in stage 11 larvae (Figure 2). If the enrichment analysis is restricted only to genes up-regulated at 11 dph, the BP terms or KEGG pathways that are found to be significantly enriched are mainly related to metabolism, particularly glucose metabolism (*e.g.* GO:0016052 ~ carbohydrate catabolic process, GO:0006096 ~ glycolysis and dre00010:Glycolysis/Gluconeogenesis). An heatmap of gene expression values across larval transitions is reported in Additional file 2.

#### Comparison of 11 and 13 dph larvae

The comparison of 11 and 13 dph larvae yielded the lowest number of differentially expressed genes, with only 120 probes significant at FDR 1%. Among the 120 transcripts, no KEGG pathways and only a few BP terms were significantly enriched. The majority of significant terms (15 of 18) were related to visual and neuronal processes; however, genes belonging to these processes displayed low fold-changes and did not exhibit an univocal trend in expression (see Figure 2).

#### Comparison of 13 and 18 dph larvae

The larval transition between 13 and 18 dph is also characterised by the significant down-regulation of all BP terms related to visual and neural processes. Up-regulated genes include those involved in muscle morphogenesis and functioning (i.e. GO:0006941 ~ striated muscle contraction, GO:0003012 ~ muscle system process, GO:0030239 ~ myofibril assembly, dre04260:Cardiac muscle contraction and dre04270:Vascular smooth muscle contraction), such as *slow myosin heavy chain 2* (N_isotig21306, 2.73 fold), *slow myosin heavy chain 3* (N_isotig01223, 2.98 fold), *titin a* (N_isotig04075, 2.18 fold) and *titin b* (N_isotig11618, 2.16 fold), which all displayed over-expression at 18 dph, with further increases over time (see Additional file 2).

#### Comparison of 18 and 24 dph larvae

Statistical analysis of the entire set of gene expression values identified a close relationship between 18 and 24 dph larvae; that in some cases (AutoSOME clustering and HCL) have also been grouped in the same cluster. However, functional analysis of differentially expressed genes identified an over-expression of genes involved in glucose metabolism (e.g. *fructose-1,6-bisphosphatase 2, glucose phosphate isomerase b* and *2,3-bisphosphoglycerate mutase*) with several BP terms (i.e. GO:0006096 ~ glycolysis and GO:0006007 ~ glucose catabolic process) more than 10-fold enriched (see Additional file 3). This finding is also supported by KEGG pathway analysis, which identified “Glycolysis/Gluconeogenesis” as the most significant term.

#### Comparison of 24 and 33 dph larvae

The comparison between 24 and 33 dph larvae identified 1,316 differentially expressed genes, with 41 significantly enriched BP terms. A total of 16 biological processes related to cell division and chromosome organisation were represented by genes that were under-expressed at 33 dph compared to 24 dph. Up-regulated genes are involved mainly in muscle cell development (10 of 41 BP terms).

### Temporal expression of “hatching” enzymes

**Figure 3:**
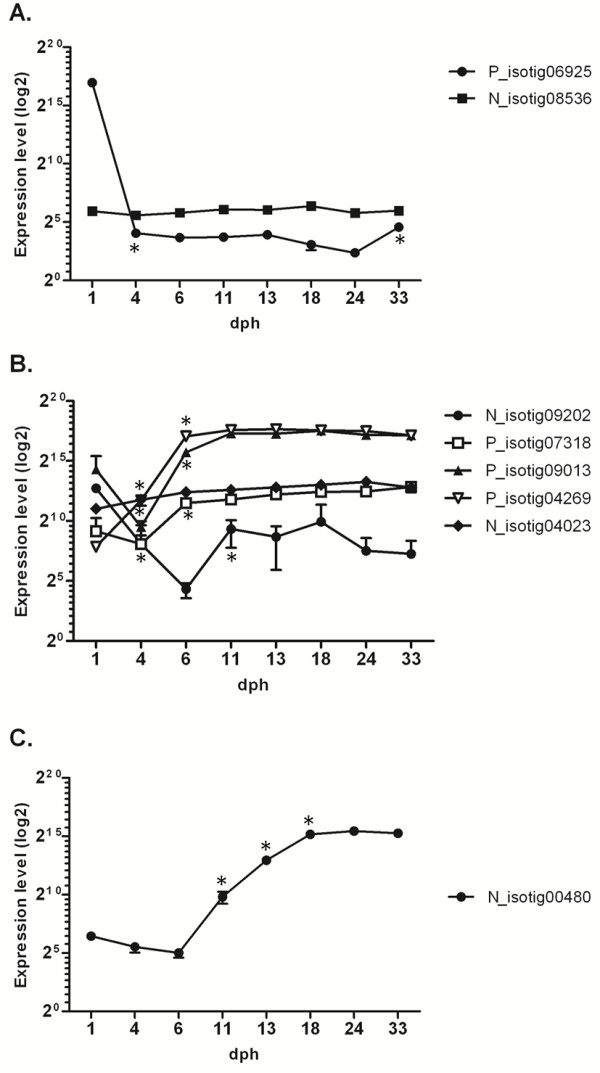
**Temporal expression of “hatching” enzymes. **Gene expression levels (log2), from 1 to 33 dph, of *S. solea *transcripts codifying hatching enzymes. **A**. *S. solea *hatching enzymes (HCE and LCE); **B**. *S. solea* homologs of astacin-like protein; **C**. *S. solea *N_isotig00480. Graphics show mean gene expression measured with microarray, bars indicate standard deviation (SD) across biological replicates. Statistical significance (p < 0.05) when comparing one larval stage against the previous one is indicated by asterisk (*).

A recurrent annotation in genes that are significantly up- or down-regulated during larval stage transitions is “hatching enzyme”. In teleosts, several genes encoding hatching enzymes have been reported. In the common sole transcriptome, eight transcripts were found to encode a putative astacin-like metalloprotease. Phylogenetic reconstruction of the evolutionary position of these protein sequences was conducted by comparison with all available astacin-like metalloproteases from vertebrate genomes (Additional file 4). Two sole sequences (P_isotig06925 and N_isotig08536) were classified as “true” hatching enzymes belonging to the groups High Choriolytic Enzymes (HCE) and Low Choriolytic Enzymes (LCE), respectively [16]. The remaining putative proteins were clustered with a large group of paralogues, which include zebrafish nephrosin and several medaka astacin-like metalloproteases. The only exception is the protein encoded by transcript N_isotig00480, which has a basal position in the phylogenetic tree (see Additional file 4). As noted previously, the majority of these transcripts were found to be significantly down- or up-regulated during stage transitions in sole larvae (Figure 3). The expression profiles for sole LCE (N_isotig08536) revealed basal expression without significant variations, while HCE (P_isotig06925) showed a dramatic decrease (>7,500 fold) from 1 dph to 4 dph, as expected for enzymes that are secreted by the embryo to degrade chorion proteins for hatching. However, P_isotig06925 displayed significantly increased expression (4.6-fold in 33 dph compared to 24 dph) after completion of metamorphosis (see Figure 3A). Five transcripts (N_isotig09202, P_isotig07318, P_isotig09013, P_isotig04269 and N_isotig04023) are putative homologs of a group of medaka astacin-like proteins that were found to be expressed in epithelial layers of internal organs (liver, intestine and kidney) in developing larvae and adults [16]. These transcripts display different patterns of expression (see Figure 3B). N_isotig04023 displays a steady increase in expression over time from 1 to 24 dph, while N_isotig09202 is characterised by mRNA levels that decrease (339.4-fold) from 1 to 6 dph, followed by a significant increase (48.7-fold) until 18 dph and a new decline thereafter. P_isotig09013 and P_isotig04269 (see Figure 3B) appear to be expressed at very high levels and are characterised by an earlier significant up-regulation (6–11 dph). The most interesting pattern, however, was observed for N_isotig00480, which displayed a steep up-regulation (>1000-fold) during the transition between 6 and 18 dph, a peak at 24 dph (stage IV, fully asymmetrical eye) and a decline at 33 dph (see Figure 3C). N_isotig00480 has no orthologues in any vertebrate genome, apart from an uncharacterised protein in stickleback and merits further study to characterise in greater detail its role during sole development.

### Expression of the TRH, TSH and TH receptors during larval development

**Figure 4:**
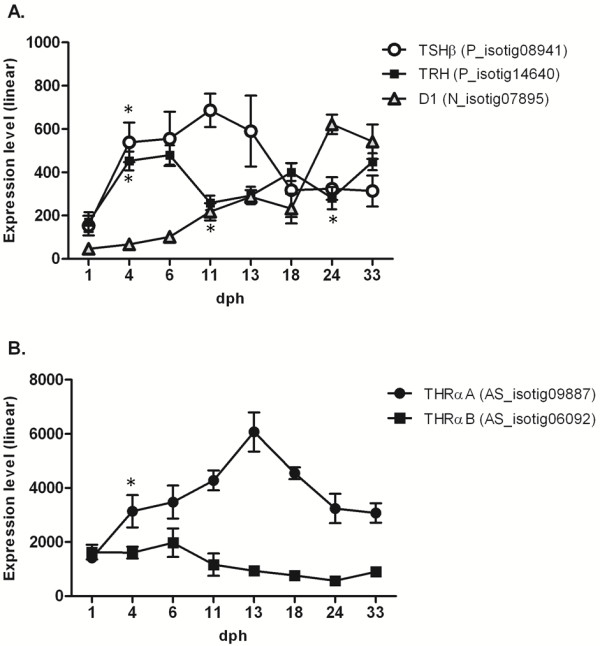
**Expression of TRH, TSH and TH receptors during larval development. **Gene expression levels (log2), from 1 to 33 dph, of *S. solea *transcripts codifying key genes of the TH cascade. **A.***S. solea *TRH, TSH and D1 transcripts; **B.***S. solea *THRαA and THRαB transcripts. Graphics show mean gene expression measured with microarray, bars indicate standard deviation (SD) across biological replicates. Statistical significance (p < 0.05) when comparing one larval stage against the previous one is indicated by asterisk (*).

In the present study, the gene expression profile of key transcripts of the TH cascade was assessed. Both *Thyrotropin releasing hormone* (TRH) and *Thyrotropin* (*Thyroid Stimulating Hormone*, TSH) can be detected very early during larval development (1–4 dph, see Figure 4A). TRH mRNA levels (P_isotig14640) increase significantly in the early stages of development, reaching a peak of expression at 6 dph (2.8 fold compared to 1 dph) after the first feeding, followed by a reduction until metamorphosis is completed. A similar trend, although shifted forward, can be observed for *Thyrotropin*, for which the TSH β transcript (P_isotig08941) displays a gradual increase in expression, with a peak at 11 dph (3.6-fold compared to 1 dph). The expression pattern of *Iodothyronine deiodinase I* (D1)*,* which controls the conversion of T4 to T3 as well as the inactive metabolite rT3, was also assessed. After hatching, D1 expression (N_isotig07895) increased gradually until the end of metamorphosis (24 dph, see Figure 4A) when it reached its highest level (~13.5-fold compared to 1 dph). THs indirectly regulate downstream gene transcription by binding to thyroid hormone receptors (TRs). In teleosts, two genes encoding TRα (referred to as TRαA and TRαB) and two TRβ have been reported [17,18]. In the present study, only *Thyroid hormone receptor α* (TRα, both TRαA and TRαB) was represented on the array; Blast searches on sole transcripts failed to detect any putative TRβ isoform. The expression profiles of the TRα genes during larval development display a particular pattern with TRαA and TRαB characterized by an opposite trend (Figure 4B). The TRαA transcript (AS_isotig09887) increases in expression until the onset of metamorphosis (13 dph), at which point mRNA levels are 4.3-fold higher than at 1 dph, followed by a decrease in expression, while TRαB (AS_isotig06092) displays a higher level of expression from 1 to 6 dph, followed by a gradual decrease until 24 dph (3.5-fold compared to 6 dph).

### Temporal expression of Growth hormone and Insulin-like Growth Factor-I system

**Figure 5:**
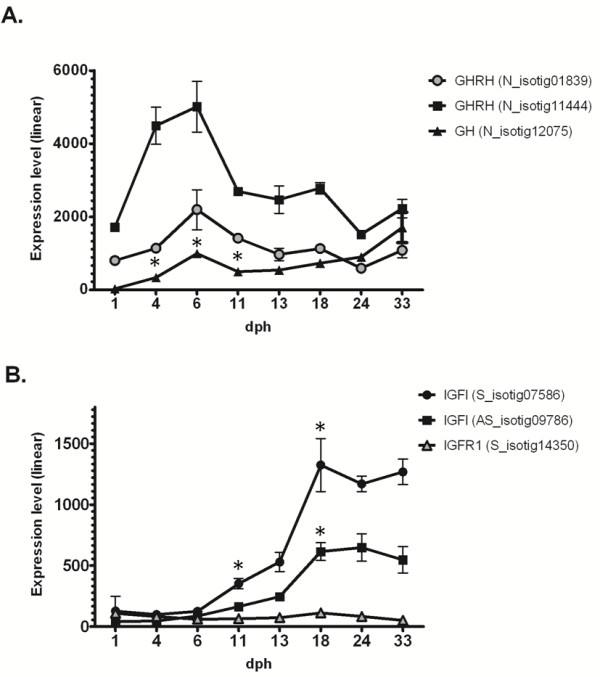
**Expression of GH/IGFI axis genes during larval development. **Gene expression levels (linear), from 1 to 33 dph, of *S. solea *transcripts codifying key genes of the GH/IGFI pathway. **A.***S. solea *GH and GHRH transcripts; **B.***S. solea *IGFI and IGFIR transcripts. Graphics show mean gene expression measured with microarray, bars indicate standard deviation (SD) across biological replicates. Statistical significance (p < 0.05) when comparing one larval stage against the previous one is indicated by asterisk (*).

In the present study, several factors belonging to the GH-IGFI “axis” were identified and their gene expression was assessed during larval development. *Growth Hormone* (GH), a protein involved in major physiological processes in the body, is characterised by a particular gene expression profile (see Figure 5A), with an increase in mRNA levels from 1 dph to 6 dph (34.5-fold), followed by a significant decrease at 11 dph (2-fold) and a subsequent gradual increase until 33 dph (with gene expression levels twice those of 6 dph). A similar pattern can be observed for *Growth Hormone Releasing Hormone* (GHRH, S_isotig11444 and N_isotig01839), although the second increase in gene expression levels began only at 24 dph (see Figure 5A). Other molecules associated with GH, such as *Growth factor receptor-bound protein 2* (GRB2, N_isotig07093 and P_isotig04733) and *GRB2-associated-binding protein 1* (P_isotig08245 and N_isotig01885), displayed no significant variation in expression across larval stages.

A completely different trend in gene expression was observed for IGFI (represented on the array by two contigs, S_isotig07586 and AS_isotig09786, that cover two non-overlapping regions of the same transcript), for which mRNA levels were relatively low during the initial stages (from 1 to 6 dph), with a gradual increase from 11 dph (see Figure 5B). At later stages (18–33 dph), gene expression levels are at least 10-fold higher compared to 1 dph (10- and 13.5-fold for S_isotig07586 and AS_isotig09786, respectively). Several *IGF-binding proteins* (IGFBPs) were also identified in the present study, with a heterogeneous assortment of expression profiles. IGFBP1, which is present as two isoforms, IGFBP1a and IGFBP1b, displayed an increase over time with a peak in expression at 33 dph (3.8- and 4.3-fold, respectively, when compared to 24 dph; 18.7- and 22.1-fold when compared to 1 dph). IGFBP2a was characterised by an initial peak at 11 dph (1.8-fold compared to 1 dph) and a second peak at 33 dph (2.3-fold compared to 1 dph), while IGFBP4 exhibited a decrease in expression at 6 dph (2-fold compared to 1 dph), followed by an increase over time until 24 dph (3.9-fold compared to 6 dph).

### Curved transcriptome landscape during flatfish development

As mentioned above, a distinctive pattern was observed in the distribution of samples along the second component (Y-axis) in the PCA (Figure 1A), in which the transcriptome landscape describes a marked curve as compared to the linear trend along the first component, which reflects temporal transitions across developmental stages. To further evaluate this observation, a SAM quantitative correlation analysis was conducted to identify genes that significantly correlated with the projected position of individual samples along the Y-axis. A total of 530 probes were positively correlated with Y-axis position (FDR 0%), *i.e.* up-regulated in 11-dph, 13-dph and, to a lesser extent, 6-dph larvae (Figure 1A), and 508 probes were negatively correlated (Additional file 5). Similar results were obtained when considering two groups of samples, one including 1, 4, 18, 24, and 33 dph larvae, the other 6, 11, and 13 dph larvae, in a SAM two-class analysis (622 up- and 524 down-regulated transcripts). Following a conservative approach, only genes that were found to be significant using both methods were considered further.

Functional annotation of all significantly up-regulated and positively correlated genes (372) using DAVID revealed that 29 GO_BP terms were significantly enriched (Additional file 6). Among the most enriched pathways, two are related to metabolism of folic acid derivatives (GO:0009396 and GO:0006760, 16- and 13-fold enriched, respectively). Genes belonging to these GO terms include *GTP cyclohydrolase 1*, whose product is a cofactor for tyrosine supply during melanogenesis [19], and MTHFD1 (*methylenetetrahydrofolate dehydrogenase (NADP + dependent) 1a*), which plays a key role in *de novo* purine and pyrimidine biosynthesis in humans [20]. Nucleotide metabolism is also over-represented, with three terms related to nucleotide catabolic processes (GO:0009166, GO:0034656 and GO:0034655) that were approximately 10-fold enriched (p < 0.05).

Several GO terms are linked to oxidative phosphorylation (e.g. P_isotig14727 *NADH dehydrogenase [ubiquinone] 1 alpha subcomplex subunit 6*, and P_isotig16393 *NADH dehydrogenase 1 alpha subcomplex subunit 11*) and glycolysis (e.g. *hexokinase 1*, *glucose phosphate isomerase a*, and *lactate dehydrogenase B*). Significant enrichment in cellular localisation was found for integral membrane proteins (Additional file 6). Concordant evidence was provided by GO terms on Molecular Function (Additional file 6). KEGG pathway analysis revealed several enriched pathways. The most relevant were the “Mevalonate pathway (dre00900)”, a cellular pathway leading either to cholesterol synthesis or to protein lipidation and “Arachidonic acid metabolism (dre00590)” (Additional file 6). Several GO terms as well as gene-specific annotations for positive/up-regulated genes suggested a putative role in liver and intestine function. To further explore this hypothesis, the list of significant genes was compared to transcripts that have been identified as specifically expressed in the developing gastrointestinal system of zebrafish larvae [21]. In that study, zebrafish larval cells were specifically sorted and analyzed using a zebrafish Affymetrix microarray platform. Of 372 sole-significant genes (Additional file 7), 161 corresponded to a putative zebrafish orthologue represented by Affymetrix probes. These genes were matched against 1,973 zebrafish genes that were found to be up-regulated in the developing gastrointestinal system of zebrafish. A highly significant overlap was found (Fisher’s Exact Test p < 0.0001), with 61 transcripts shared between the two sets. Also of note is the presence of several genes involved in the scavenging of oxygen radical species (*e.g. superoxide dismutase 1*, *glutathione peroxidase* and *glutathione S-transferase*) and mitochondrial carriers.

Two genes involved in retinol metabolism were also included in the list of significant transcripts. *Bcox,* a provitamin A-converting enzyme with a role in zebrafish embryogenesis and pigmentation [22], displayed the most striking profile, with a peak at 13 dph. The second protein was *retinol binding protein 2* (RBP2), an intracellular chaperone for retinol and retinal, which is involved in the intestinal absorption of vitamin A as well as in modulating the supply of retinoic acid in specific cell types. RBP2 displays a different expression profile, with an initial peak at 6 dph and a second, less marked, at 13 dph. Notably, among the up-regulated and positively-correlated genes was a key regulator of morphogenesis (TSH-beta, P_isotig08941). A smaller set of genes (190) were found to be down-regulated as well as negatively correlated with the second PCA component (Additional file 8). Functional annotation with DAVID highlighted Tight junction and Cell-cell adhesion as KEGG pathways significantly enriched. Members of these pathways include *claudin 4* (P_isotig13499), *claudin 7b* (N_isotig12365) and *occludin b* (P_isotig18663), all significantly down-regulated on 6-11-13 dph compared to previous and later stages. In this context, *vitronectin a* (P_isotig17782, 2.9- and 3.9-fold down-regulated compared to 1 dph and 33 dph, respectively), *integrin, alpha-V* (N_isotig20767, 2.3- and 1.4-fold down-regulated compared to 1 dph and 33 dph, respectively) and *calpain-3* (N_isotig19399, 5.2- and 6.4-fold down-regulated compared to 1 dph and 33 dph, respectively), genes that play a role in focal adhesion and ECM-receptor interactions, were also identified as significant.

### Real-time RT-PCR validation

A set of 10 genes was assessed by RT-qPCR to validate the microarray platform performance. This set of genes was chosen among those involved in key pathways of *S. solea* larval development and displaying, in the present study, different patterns of expression across stages.

Three genes, Bcox, RBP2 and *claudin 7b*, were correlated (negatively or positively) to sample projection on the PCA Y-axis and were either down- (*claudin 7b*) or up-regulated (Bcox and RBP2) in pre-metamorphic larvae compared to previous and later stages. Three genes, *megalin*, *sonic hedgehog-like* and *aldolase a*, are members of key pathways of larval development (“Hedgehog signalling” and “Glucose metabolism”) and displayed expression levels that were positively (*aldolase a*) or negatively (*megalin* and *sonic hedgehog-like*) correlated with time of larval development. The latter four genes, TSHβ, TRH, GH and IGF1, are members of the TH and GH/IGF1 cascades and displayed different patterns of expression across larval stages.

A Spearman rank-correlation test was used to assess the correlation between expression values measured by RT-qPCR and by microarray in a total of 16 experiments (see Methods). Table 2 indicates the correlation coefficients calculated for the validated genes. All genes displayed high correlation coefficients between microarray and RT-qPCR, with Spearman’s rho 0.75 < rho < 0.95 (p < 0.01), thus confirming a strong positive correlation between the two technologies.

**Table 2:** Correlation between microarray and real-time RT-PCR expression data

**Sequence name**	**Gene name**	**Spearman’s rho qPCR/probe**
**Contig00403**	*Aldolase A*	0.946**
**Isotig07996**	*Megalin*	0.845**
**Isotig04483**	*Bcox*	0.946**
**Isotig14204**	*Retinol binding protein 2*	0.925**
**Isotig12075**	*Growth hormone*	0.760**
**Isotig07586**	*Insulin growth factor I*	0.943**
**Isotig08941**	*Thyroid Stimulating Hormone*	0.950**
**Isotig14640**	*Thyrotropin releasing hormone*	0.811**
**Isotig18139**	*Sonic hedgehog-like*	0.811**
**Isotig12365**	*Claudin 7b*	0.750**

**p < 0.01.

## Discussion

The continuous development of advanced next-generation sequencing (NGS) technologies and their competitive costs have resulted in their use for a wide range of applications, included genome and transcriptome sequencing of non-model species. To date, only limited transcriptome-wide studies on fish larvae have been conducted in teleost species [23-26], and only one study has been conducted in flatfish (*i.e.* Atlantic halibut [27]), a group of great interest because it undergoes a dramatic larval-to-juvenile transition compared to other fish species. The present study is the first to characterise gene expression profiles during metamorphosis of *Solea solea*, a flatfish species of relevance for Mediterranean aquaculture. The use of 454 pyrosequencing produced a large set of good quality reads that were then assembled into a significant number of Isotigs. The combined approach used in this study allowed the annotation of a high percentage (>75%) of sole Isotigs, considerably higher than the value observed in similar studies on other fish species (turbot 50.7% [28], Senegalese sole 40.6% [10], pre-smolt Atlantic salmon 50.3% [29], channel catfish 51% [30], European eel 33% [31], gilthead seabream 66% [26]). Annotated transcripts were then employed for the construction of an oligo-DNA microarray in order to investigate the transcriptome variation during *S. solea* larval development. The variety of biological processes that were found to be differentially expressed between time points reveals the complexity of transcriptome regulation during larval ontogenesis. Changes in gene expression reflect processes related mainly to organogenesis (*e.g.* muscular development, visual and neural development and ossification) and the maturation of essential physiological functions (*e.g.* digestive function and metabolic pathways). Analysis of differentially expressed genes during stage transitions delineated the main processes involved in organogenesis and larval growth.

Development of visual perception is essential for larval feeding. The eye of indirectly developing species is often slightly pigmented or non-pigmented at hatching and is most likely non-functional. In most species, the eye becomes fully pigmented and functional within the first week after hatching [32]. Functional annotation of differentially expressed genes revealed a strong up-regulation of BPs related to visual perception during the early stages of larval development (see Additional file 3). Many genes associated with eye morphogenesis were found to be up-regulated until 6 dph, while an opposite trend was observed from 11 dph and thereafter, suggesting that this stage represents the turning point for eye development. Pitx2 (*Paired-like homeodomain transcription factor 2*, N_isotig16834), a gene involved in anterior eye segment development, displayed higher expression levels at 1 and 4 dph. *Opsin* (*i.e.* green-, blue- and red-sensitive opsins) and *rhodopsin* transcripts, primary light sensors of retinal photoreceptors [33,34], as well as *retinal arrestin*, which is involved in signal transduction, displayed a strong up-regulation at 4 and 6 dph. In the literature, all these genes are reported to be regulated by vitamin A [27,35-37]. Vitamin A (Retinol) is indispensable for eye morphogenesis [38] and exerts its biological function through its metabolite, retinoic acid (RA), a signal molecule important for photoreceptor development in vertebrates. In the present study, KEGG pathway analysis highlighted “Retinol metabolism” (dre00830) as significantly enriched (p < 0.05, Fold enrichment >3) at 4 and 6 dph. Many genes that play a crucial role in RA synthesis and transport, such as RDH10 (*Retinol dehydrogenase 10*), RALDH2 (*Retinaldehyde dehydrogenase 2*), CRABP (*Cellular retinoic acid binding protein 1*) and CRBP (*Cellular retinol-binding protein*), displayed high levels of expression at the earliest larval stages, followed by a decrease over time, supporting the hypothesis that RA is mainly produced in specific temporal windows [39]. Retinoic acid treatment influences photoreceptor development in zebrafish and has been reported to up-regulate the transcription of several photoreceptor-specific genes [36], as it was observed for opsins and arrestin in the present study. Through *in situ* hybridisation of different opsin cDNAs on single cones expressing either blue-, green- or red-sensitive opsin, Prabhudesai and colleagues [36] demonstrated that, in zebrafish, exposure to endogenous RA differentially regulates gene transcription on specific photoreceptors with a simultaneous increase in the expression of rod opsin and red cone opsin and a decrease in the expression of blue and UV cone opsins. The results obtained in the present study were not in agreement with this evidence, as both red-, green- and blue-sensitive opsins were up-regulated at 4 and 6 dph, with green-sensitive opsins displaying the highest expression levels (~10-fold). However, this inconsistency may be due to the methods applied for assessing the expression of different opsin sets because, to be comparable, the microarray analysis should have been performed on single photoreceptors already committed to a specific phenotype rather than globally. Taken together, all the above evidence clearly indicate that genes involved in the ontogenesis of the visual system are up-regulated during early stages of larval development (4–11 dph), further confirming that the eye is still developing during this larval period.

Many genes related to muscle development and function increased over time (see Table 1 and Additional file 2), and the corresponding BPs were significantly enriched (up to 10-fold) during the transition from 13 to 18 dph larvae and later stages. C*aveolin 3* is involved in zebrafish muscle precursor differentiation, and *Cav3* knockout mice exhibit a mild muscular dystrophy phenotype [40]. The transcription factor *myocyte enhancer factor-2* (MEF2) is important for all types of embryonic muscle differentiation and, in Drosophila, plays an essential role in adult myogenesis by participating in the control of myoblast fusion and in myofibrillogenesis in developing myotubes [41]. *Galectin-1* promotes skeletal muscle differentiation in human foetal mesenchymal cells [42] and has been shown to be involved in muscle regeneration. *Titin a* is required for sarcomere assembly and is considered a molecular marker for cardiomyocyte differentiation [43]. These data are in agreement with those obtained for the European seabass [23] and the Atlantic halibut [27] and reflect the progressive development of muscle during larval ontogenesis. Post-embryonic growth is associated with both hypertrophy (increase in muscle fibre diameter) and hyperplasia (recruitment of new muscle fibres) from undifferentiated myoblasts or myosatellite cells [44]. Gene expression analysis alone is not sufficient to discriminate between those two mechanisms of muscle growth without the support of other analyses (*i.e*. histology), however our findings are in agreement with previous studies conducted in fish [3] where it is reported that the mechanism of muscle development during fish larval growth is mainly related to stratified hyperplasia, which is responsible for the increase in number of muscle fibers.

A clear correspondence was also observed between muscle development and energy metabolism. Energy metabolism in early fish larvae is almost entirely aerobic. The anaerobic power of muscle fibres is low after hatching but increases during the transition from larva to juvenile [3]. Fish larvae primarily use dietary lipids and proteins as their first energetic sources. This is reflected by the sole gene expression profiles, in which many genes involved in proteolysis and lipid metabolism are up-regulated at the mouth-opening stage (6 dph). This energy production pathway is masked in later stages by the increase in the expression of genes involved in glucose metabolism (Table 1), with several BP terms and KEGG pathways significantly enriched at 24 dph. Of particular interest, the gene expression of *lactate dehydrogenase* (LDH), the enzyme that catalyses the interconversion of pyruvate and lactate when oxygen is absent (or in short supply), increased gradually, with fold-changes of 29-fold (at 24 dph) and 58-fold (at 33 dph) for LDH-A (P_isotig00860) and LDH-B (N_isotig20169), respectively. This finding further confirms a previous study by Darias and coworkers [23] that suggested a relationship between glucose metabolism and the development of white muscle fibres, in which the anaerobic energy necessary for swimming is mainly supplied by the glycolytic pathway.

### Coordinated expression of the TH cascade during larval metamorphosis

Thyroid hormones (TH) are among the most prominent factors controlling vertebrate development and metabolism in adult animals. In particular, 5′,3′,5,3-tetraiodothyronine (T4) and 3′,5,3-triiodothyronine (T3) are widely reported to play a key role in larval development and fish metamorphosis [17,45-47]. Flatfish metamorphosis is a TH-driven process, and numerous studies have demonstrated the importance of these hormones for metamorphosis-associated tissue modifications [45-48]. The start of metamorphosis has been associated with a surge in THs in which TH levels increase until the metamorphic climax and decrease post-climax [46]. Japanese flounder (*Paralichthys olivaceus*) and Atlantic halibut (*Hippoglossus hippoglossus*) metamorphosis has been proven to be induced precociously by TH treatment and, conversely, to be delayed or abolished following exposure to agents that inhibit TH synthesis [17]. Several studies have assessed the expression profiles of genes belonging to the thyroid-pituitary axis in flatfish, although focusing on a single gene or a few genes without a global view of the whole gene network. In the present study, both TRH and TSHβ transcripts were detected; however, these genes exhibited slightly different expression patterns during development. TRH reached its maximum of expression at 6 dph, while the TSHβ peak was shifted forward to 11 dph. These findings are in good agreement with those previously reported for the Senegalese sole [17] and other fish species [45]. Temporal regulation of mRNA levels is coherent with the role of TRH, which promotes the synthesis and release of TSH. TSH in turn acts on the thyroid follicles by inducing iodothyronine deionidase activity [49], which ultimately leads to the synthesis of T4 and T3, which display higher concentrations at the climax of metamorphosis. Only slight changes in *Iodothyronine deionidase I* (D1) expression occurred during the pre-metamorphosis stages, with an initial peak in expression at 13 dph (onset of metamorphosis) and strong up-regulation at 24 dph (metamorphosis climax). D1 has been reported to play a double role in TH synthesis by i) activating T4 by producing the more active T3 and ii) inactivating T4 by producing the metabolite rT3 [46]. The peak of expression observed at 24 dph appears to be in better agreement with the inactivating function of D1, with a possible role in decreasing circulating levels of THs after metamorphosis.

Higher vertebrates commonly possess two principal TR isoforms, namely, TRα and TRβ, and two genes encoding TRα (TRαA and TRαB) have been found in different fish species [18,50-52] as a result of the whole genome duplication that occurred during teleost evolution [53]. To date, two TRβ genes have been reported only in *Conger myriaster*[54]. In this study, two transcripts encoding TRα genes were identified, and their expression in common sole is similar to what has been reported during metamorphosis in halibut [18] and gilthead seabream [45] and suggests a correlation between TH levels and TRαA. This evidence supports the hypothesis postulated by Galay-Burgos [18] that TRαA is itself a TH-inducible gene.

### Role of the GH/IGFI system during sole larval development

The Growth Hormone (GH) - Insulin-like Growth Factor-I (IGF-I) system is widely recognised for its importance in the regulation of growth and development in fish by participating in the determination of both physiological processes and behavioural aspects [55]. During the last decade, there has been a growing interest in the physiological role of the GH/IGF system in developmental processes of fish. Although several studies have investigated the role of key components of the GH/IGFI axis in fish growth [55-57], there is no information on the temporal regulation of corresponding mRNA levels during larval development and metamorphosis. In the present study, analysis of the mRNA levels of GH, GHRH, IGFI, IGF1R and IGFBPs revealed a trend in expression coherent with their postulated role in the GH pathway. The cascade of events by which GH induces its biological function begins when GH, whose expression is stimulated by growth hormone-releasing hormone (GHRH), binds to specific GH receptors (GHR) and in turn stimulates IGFI synthesis in the liver [58].

In the Atlantic halibut, the GH-IGFI system is established at the start of autonomous feeding [59]. In the common sole, GHRH and GH display similar expression patterns, with a significant up-regulation before metamorphosis onset (6 dph, soon after mouth opening), followed by a decrease in mRNA levels until 24 dph and a then subsequent increase (albeit not significant) at 33 dph. Up-regulation of IGFI and IGFR1 appears to be concurrent with the decrease in GH mRNA levels. This evidence is in agreement with the hypothesis that IGFI synthesis acts as a mediator of a negative feedback mechanism by inhibiting GH transcription as already demonstrated in different fish species (e.g. *Oncorhynchus mykiss, Oreochromis mossambicus, Anguilla anguilla)* ([60] for a review). Other key components of the GH/IGFI pathways are IGFBPs. In the present study, IGFBP mRNAs displayed lower expression in pre-metamorphosis stages, then gradually increased and reached a peak at metamorphosis climax (24 dph, IGFBP4) or later (33 dph, IGFBP1, IGFBP2a). In the Japanese flounder, there is evidence that IGFBPs play an important role in regulating the activity of IGFs during larval development and metamorphosis [61]. In zebrafish *in vivo* expression of IGFBP1 has been reported to cause growth and developmental retardation [62]. In the common sole, down-regulation of IGFBP-1 mRNA and increased expression of IGFI mRNA were observed during metamorphosis, followed by an inversion of this trend after metamorphosis. This observation is in agreement with the need for IGFI-mediated stimulation of pre-metamorphic larval growth and initiation of metamorphosis; once metamorphosis is completed, IGFI function is negatively regulated via a reduction in its mRNA levels and sequestration of circulating IGFI by IGFBPs.

The potential regulatory effects of TH on IGFI expression remain to be fully clarified. In fish, T3 and T4 have been shown to induce hepatic IGFI expression. In zebrafish and tilapia, T3 induces IGFI transcription both *in vitro* and *in vivo*[63] and in mice, T3, in the presence of THRα, binds to a TH response element in intron 1 of the IGFI gene to stimulate its transcription [64]. These phenomena are in agreement with the gene expression profiles of both *S. solea* IGFI transcripts, which show a significant increase in mRNA levels at 13–18 dph, simultaneous with a peak in THRαA expression. In the present study, however, TH levels were not assessed.

Morphological modifications that subtend eye migration during flatfish metamorphosis are controversial. Saele and coworkers [65] propose that bone remodelling and fibroblast proliferation drive Atlantic halibut eye migration, while a recent study by Bao et al. [66] assumes that in different fish species (i.e. *Solea senegalensis, Cynoglossus semilaevis, P. olivaceus*) the initial migration of the eye is caused by cell proliferation in the suborbital tissue of the blind side and that the twist of frontal bone is dependent on eye migration. Recent studies ([58] and references therein) have proposed that the GH-IGFI system participates in cranial remodelling during halibut metamorphosis as key tissue/cell types (*e.g.* muscle, fibroblasts, etc.) have been proven to be sensitive to GH and IGF1 during metamorphosis. The expression profiles of sole genes involved in the GH/IGFI pathway, particularly IGFI and IGF1R, appear to support such a hypothesis, despite the fact that, in the present study, transcriptomic data were obtained from whole larvae without tissue-specific information.

### Transcriptomic landscape in pre-metamorphic larvae

Although the initiation of lateral asymmetry and eye migration was only observed in 13 dph larvae of common sole, it is likely that transcriptional changes occur before phenotypic modifications become evident. In this respect, PCA analysis clearly indicated a specific clustering along the second component for 6, 11 and 13 dph larvae, compared to all earlier and later developmental stages. Considering the large number of differentially expressed transcripts, it is likely that key transcriptomic events occur between 6 and 13 dph. The majority of significant genes appear to be related to the development of epithelial layers and digestive organs, a process that might be only indirectly involved in metamorphosis. However, a few enriched KEGG pathways that were observed in the same stages are suggestive of additional developmental processes. The “Mevalonate pathway”, which was found to be significantly enriched in up-regulated genes, leads either to cholesterol synthesis or to protein lipidation, both processes that are relevant during development (e.g. several small GTPases require prenylation for activity). Similarly, the significant enrichment of the “Arachidonic acid pathway” and, in particular, *prostaglandin E2 (PGE2)-synthase* can be linked to developmental processes, as PGE2 has been shown to be involved in the morphogenesis of several organs.

As mentioned previously, the molecular mechanisms underlying flatfish metamorphosis are still under debate, and eye migration and external asymmetry are apparently a separate process from somatic growth and organogenesis [65]. A recent study [67] hypothesised that flounder eye-sidedness is controlled by the *nodal-lefty-pitx2* (NLP) pathway, in which pre-metamorphic Pitx2 re-expression on the left side habenula drives eye lateralisation by stimulating cell proliferation. In the present study, Pitx2 (N_isotig16834) displayed reduced mRNA levels over time, with no peaks in pre-metamorphic stages. However, it should be emphasised that gene expression was assessed in whole larvae (without separation of the right and left sides of the animal), and the observed decrease in expression could arise from the decrease in habenula/whole body tissue mass ratio that occurs during larval development. Bao and colleagues [66] reported that initial migration of the eye in flatfish is caused by fibroblast proliferation in the suborbital tissue of the blind side. Once the eye receives sufficient pushing force from proliferating cells to overcome the main counteracting force from the other eye, it begins migrating upwards. Single-cell and collective migration are known to occur during morphogenesis, tissue regeneration and in pathological conditions. In particular, collective cell migration is essential in building, shaping, and remodelling complex tissues and tissue compartments. Cell–cell and cell–matrix adhesion, cytoskeletal polarity and rigidity, and pericellular proteolysis interdependently control migration mode and efficiency [68]. Interactions of cells with the extracellular matrix (ECM) are essential for the control of tissue remodelling and cell migration because the ECM provides the substrate as well as a barrier towards the advancing cell body. Motility is limited by the turnover rates of adhesion and de-adhesion events, yielding an inverse relationship between contact strength and migration rates. At the cell-extracellular matrix contact points, specialised structures known as focal adhesions are formed, in which actin filaments are anchored to transmembrane receptors of the integrin family. Focal adhesion can be controlled by modulation of integrin expression levels, degradation of ECM structures by proteases, and focal contact disassembly by cytoskeletal reorganisation (reviewed by [69]). In this context, interesting findings arose from the identification of genes that were differentially expressed in pre-metamorphic *S. solea* larvae. Functional annotation and KEGG pathway analyses revealed that “Focal adhesion” (dre04510) and “Tight Junction” (dre04530) were differentially expressed in pre-metamorphic stages and 18 dph larvae. *Vitronectin a* (P_isotig17782), a cell adhesion factor found in serum and ECM that is recognised by certain members of the integrin family and serves as a cell-to-substrate adhesion molecule, was found to be down-regulated in 6, 11 and 13 dph larvae compared to previous and later stages. Several components of tight junctions displayed a similar pattern of expression. Members of the claudin family (*i.e. claudin* 5b, 8d, 15d and 30c), which in some cases have been reported to have a role in adhesion to ECM and inhibition of cell motility in human cancer [70], were under-expressed in pre-metamorphic stages. Similar evidence was observed for the transcript encoding *occludin b* (P_isotig18663).

An opposite trend was observed for *matrix metalloproteinase-2* (MMP2) and collagenase MT1-MMP, both reported by Friedl and coworker [69] to play a key role in ECM degradation during cell migration. MMP2 was up-regulated at 11-18-24 dph, while MT1-MMP was over-expressed at 18–24 dph. Another group of metalloproteases that is potentially involved in pre- and early metamorphosis stages is that represented by astacin-like metallo-endopeptidase. Astacin-like zinc-metalloproteases, also known as hatching enzymes, are widely present in egg-laying animals and play a key role in degrading chorion proteins to release the developing embryo. As reported above, a significant increase in expression from 6 dph was observed for three members of this family, encoded by transcripts N_isotig09202, P_isotig09013 and P_isotig04269 (see Figure 3B and Results). Evolutionary analysis of astacin-like metalloproteases in the medaka and zebrafish genomes [16] demonstrated that one or two genes represent *bona fide* “hatching enzymes”, while the rest are paralogous copies of still unclear function. A recent study [71] revealed that these proteins are involved in mouse and chicken embryogenesis and organogenesis, mostly in remodelling epithelia. Whether astacin-like metallo-endopeptidases have a similar role in the common sole and, more specifically, in promoting cellular/tissue migration through ECM reorganisation during early development represents an interesting topic for further studies.

Finally, coordinated changes in the actin cytoskeleton as well as the actin-myosin interaction provide the force for cellular motility. In the present study, both α-actin and myosin light-chain displayed increased levels of expression at 13 dph and thereafter. The over-expression of these genes could be simply related to muscle development, which occurs during the same stages; however, a role in molecular mechanisms of cell migration and, more generally, in tissue rearrangement cannot be excluded.

In conclusion, despite the limitations of gene expression analysis on whole larvae, including the absence of cell- or tissue-specific information and the potential signal dilution of scarcely represented transcripts, the characterisation of the larval sole transcriptome permitted a global view of the main molecular mechanisms underlying physiological and morphological changes during the larval-to-juvenile transition. In particular, the observation of a peculiar shape in the transcriptomic landscape of 6–13 dph larvae and the analysis of genes involved in such phenomenon indicated molecular pathways and gene networks that might be critical for the profound phenotypical changes that occur during flatfish metamorphosis.

## Conclusions

With the advent of novel methods for high-throughput DNA sequencing such as 454 pyrosequencing technology, genomic resources are gradually becoming more affordable for the study of non-model species for whom this type of knowledge remains limited. The development of different ‘omic’ technologies is thus enhancing the knowledge of the complex genetic control underlying different physiological processes of flatfishes. In the present study, the transcriptome of the common sole, a flatfish species of great interest for European aquaculture, was sequenced for the first time, and a microarray platform for gene expression profiling is now available. Gene expression analysis of developmental stages permitted the delineation of the main mechanisms underlying physiological and morphological changes during the larval-to-juvenile transition. The large variety of biological processes found to be differentially expressed between time points reveals the complexity of transcriptome regulation during larval ontogenesis. The detailed analysis of the transcriptomic landscape of pre-metamorphic larvae indicates molecular pathways and gene networks that might be critical for the profound phenotypical changes that occur during flatfish metamorphosis.

## Methods

### Larval rearing and sampling

Common sole larvae came from one batch of fertilised eggs obtained from spontaneous spawning of a broodstock maintained at the Laboratory of Aquaculture, Department of Medical Veterinary Sciences, University of Bologna, Italy. Broodstock fish were captured in the Adriatic Sea and adapted to captivity over the past 6 years. Eggs and newly hatched larvae were maintained in a single incubator until mouth opening at 4 days post hatching (dph) and then allocated in three flat-bottom 280-l square tanks (2000 larvae tank^-1^). Larvae were fed according to standard hatchery feeding protocols consisting of live feed (*Artemia* nauplii until 9 dph and subsequently enriched metanauplii) with dry feed (AgloNorse, size of 150-350 μm, Ewos, Norway) as co-feed until 27 dph. *Artemia* nauplii and metanauplii were manually administered twice a day (10:00 am and 4:00 pm). Dry feed was supplied by belt feeders for 16 h day^-1^ (from 10:00 am until 2:00 am) to apparent satiation, ranging from 4 to 7 g tank^-1^ day ^-1^. *Artemia* cysts (Great Salt Lakes, Catvis BV, The Netherlands) were incubated and hatched in seawater (salinity 25 g/l) at 28 °C over 18 h. *Artemia* metanauplii were harvested and enriched for 24 h using Algamac-3050 (Aquafauna, Bio-Marine Inc. Hawthorne, USA). The tanks were supplied with natural seawater and connected to a recirculating system. The water temperature was maintained at 18.0 ± 1.0 °C, and the photoperiod was maintained at a 16/8 h light/dark. The oxygen level was 7.5 ± 1.0 ppm. Animals were reared until 33 dph, during which survival and growth were comparable to that observed in previous studies [4]. Larvae were randomly sampled at 1, 4, 6, 8, 11, 13, 18, 20, 24 and 33 dph. The onset of metamorphosis occurred at 13–14 dph (start of left eye migration) and ended at 24–25 dph (completion of left eye migration and visibility of left orbital arch on the dorsal side). Sampled larvae were sacrificed by anaesthetic overdose. The larvae were placed in a bath of 2-phenoxyethanol solution at a concentration of 0.5–0.6 ml/l until death was achieved, rinsed with distilled water and then preserved in RNA*later* (Ambion^®^, Life Technologies Ltd, Paisley, UK ) until further processing. In addition to the larvae, five wild adult soles (average weight: 132.8 g ± 13.7) from the North Adriatic Sea were also sacrificed according to the same procedure. The intestine and liver were isolated from each fish, and a portion of the collected tissues was sampled and preserved in the same way as the larvae. The method of euthanasia and all experimental procedures were evaluated and approved by the Ethical-scientific Committee for Animal Experimentation of the University of Bologna in accordance with the European Community Council directive (86/609/ECC).

### Larval staging

Eight developmental stages, from hatching until completed metamorphosis, were used in the present work to characterise the larval transcriptome of *S. solea*. At 4 dph, larvae were symmetrical, and the yolk sac was still present. Eyes were not completely pigmented, and melanophores were distributed in the finfold that surrounds the larval body. At 6 and 11 dph, larvae were still symmetrical, the mouth was open, and eyes were fully pigmented. Digestive tube development was also appreciable. At 13 dph, body asymmetry and eye migration started (Stage I as in [72]). Pigmentation covered the body, and a primordial caudal fin was visible. Larvae at 18 dph were middle metamorphic (Stage II and III as in [72]) with the left eye positioned upwards and partially visible from the right side. At 24 dph, the larval body was asymmetric. Eye translocation was complete, with the left eye on the right side and the orbital arch completely visible (Stage IV as in [72]). At 33 dph, metamorphosis was complete, and larvae appeared in their juvenile form.

### RNA extraction, cDNA library construction and sequencing

Total RNA was extracted from pools of *S. solea* larvae and adult tissues using the RNAeasy Mini Kit (Qiagen, Hilden, Germany) according to the manufacturer’s specifications. The larval pool from 1 to 13 dph contained approximately 10 individuals, while 18 to 33 dph pools contained 5 larvae each. For each sample, the RNA concentration was determined with a NanoDrop^®^ ND-1000 UV–vis spectrophotometer (NanoDrop Technologies, Wilmington, USA). RNA integrity and quality were then estimated with an Agilent 2100 Bioanalyzer (Agilent Technologies, Palo Alto, CA), and the RNA integrity number (RIN) index was calculated for each sample. Only RNAs with a RIN number > 8.5 were further processed.

A normalised cDNA library was constructed by pooling equal amounts of RNA from eight larval developmental stages (1, 4, 6, 8, 11, 13, 20, and 33 dph, one pool of larvae for each stage) and two tissues (liver and intestine) from adult specimens. cDNA library construction was performed by Evrogen JSC (Moscow, Russia); briefly, total RNA was used for double-stranded cDNA synthesis using the SMART approach. SMART-prepared amplified cDNA was normalised using the DSN-normalisation method [73]. The normalisation procedure included cDNA denaturation/reassociation, treatment with a duplex-specific nuclease (DSN, [74]), and amplification of the normalised fraction by PCR. Approximately 5 μg of the normalised cDNA library was then used for sequencing using Roche 454 FLX Titanium technology at the BMR Genomics SRL (Padova, Italy).

For gene expression profiling of larval development by DNA microarray, pooled samples of 5–10 individuals (depending on larval age) were sampled and RNA was extracted at 1, 4, 6, 11, 13, 18, 24 and 33 dph. The number of biological replicate pools was four for each sampling point.

### Read production and assembly

Sequencing was performed with GS FLX Titanium series reagents and one single region on a Genome Sequencer FLX instrument. Bases were called with 454 software by processing the pyroluminescence intensity for each bead-containing well in each nucleotide incorporation. A total of 909,466 sequence reads were produced from the normalised cDNA library constructed using a mixture of larval and adult tissues (see above). All Roche 454 FLX reads were trimmed to remove adapter sequences and have been deposited in the NCBI Sequence Read Archive (SRA) [75] under accession number SRA058691. An additional set of 314,486 reads was available from a second cDNA library of skeletal muscle (L. Bargelloni, unpublished data). In addition, 21 mRNA sequences for *S. solea* were available in NCBI [76] (as of 1st September 2011). All 454 sequence reads and all mRNAs were then assembled with Newbler 2.6 software using default settings. Newbler software produces “contigs”, “Isotigs” and “Isogroups”. An Isogroup is a collection of contigs containing reads that imply connections between them. An Isotig is meant to be analogous to an individual transcript; different isotigs from a given Isogroup can be inferred splice-variants. Ideally, Isogroups are transcripts, isotigs are splice variants of one transcript and contigs are separate exons.

### Transcriptome annotation

The Basic Local Alignment Search Tool (BLAST) was used to annotate *S. solea* Isotigs and contigs. Blast2GO software [77] was used to perform Blastn (cut off E-value of < 1.0 e-7) searches against the NCBI nucleic nr database as well as Blastx (cut off E-value of < 1.0 e-5) searches against the NCBI amino acid nr database and SWISSPROT database. By using this approach, Gene Ontology (GO) terms associations for “Biological process”, “Molecular function” and “Cellular component” were also obtained for transcripts with a significant match with a known protein. To improve the number of annotated transcripts, two additional approaches were attempted: i) blastx (cut off E-value of < 1.0 e-5) and blastn (cut off E-value of < 1.0 e-7) searches against proteins and high-quality draft transcriptomes of *Danio rerio, Gasterosteus aculeatus, Oryzias latipes, Takifugu rubripes, Tetraodon nigroviridis, Homo sapiens,* and *Mus musculus* available on the Ensembl Genome Browser (release 56, [78], ii) blastn search (cut off E-value of < 1.0 e-7) against *D. rerio, O. latipes, Gadus morhua, G. aculeatus, Ictalurus furcatus, I. punctatus, Salmo salar, Oncorhynchus mykiss, Oreochromis niloticus, Pimephales promelas, H. sapiens, M. musculus* databases stored in the NCBI UniGene database [79].

Annotated transcripts were then further clustered through mapping against a single species proteome, *i.e.* looking for independent isotigs or contigs that putatively encoded the same protein (named “redundant” for brevity). Two or more Isotigs/contigs were considered clustered together when they displayed the same annotation with at least 3 of 5 fish species when considering the Ensembl Gene IDs of five fish species (*D. rerio*, *G. aculeatus*, *O. latipes*, *T. nigroviridis*, and *T. rubripes*). In the case of two transcripts encoding the same protein, only the longer one was used for microarray design.

### Solea solea oligonucleotide microarray

Gene expression analyses were performed with the Agilent-036353 *Solea solea* oligo microarray (GEO accession: GPL16124). All unique annotated transcripts (15,385; see Results), excluding those annotated only with Unigene (2,549), were employed for microarray design. Transcript matches with ENSEMBL protein or transcript databases were then exploited to infer sole sequence orientations by identifying i) transcripts with unequivocal orientation (sequence frame concordant across all matches), ii) transcripts with ambiguous orientation (sequence frame not concordant across matches), and iii) transcripts with unknown orientation (transcripts whose match was against the NCBI nr nucleotide database). One probe for annotated sequences with unequivocal orientation (10,987) was designed while, whenever possible, two probes with both orientations (sense and antisense) were designed for Isotigs with ambiguous/unknown orientation (1,849). A total of 14,674 oligonucleotide probes (60 nt) representing 12,836 transcripts were *in situ* synthesised onto the array using Agilent non-contact ink-jet technology (8 × 15 K format, including default positive and negative controls).

A single dye (Cy3) labelling scheme was implemented, and a mixture of 10 different viral poly-adenylated RNAs (Agilent Spike-In Mix) was added to each RNA sample to monitor labelling and hybridisation quality as well as microarray analysis work-flow. Sample labelling and hybridisation were performed as reported in Ferraresso et al. [80] with slight modifications. Processed slides were scanned at 5 μm resolution with an Agilent G2565BA DNA microarray scanner. Default settings were modified to scan the same slide twice at two different sensitivity levels (XDR Hi 100% and XDR Lo 10%). The two linked images generated were analysed together, and data were extracted and background subtracted using the standard procedures contained in Agilent Feature Extraction (FE) Software version 9.5.1.

### Statistical analyses

The normalisation procedure was performed using R statistical software [81]. Microarray data were quantile normalised across all arrays. To exclude poor-quality probes from statistical analyses, hybridisation success and mean fluorescence for each probe were evaluated in a total of 31 experiments (four biological replicates for each developmental stage with the exception of 13 dph, for which one biological replicate was discarded). Microarray probes were considered unreliable when a successful hybridisation (“glsFound” equal to 1) in less than 50% of the experiments and a mean fluorescence below 10 were observed. Using this approach, 753 probes were filtered out, leaving 13,921 probes for all further analyses. A total of 546 probes of 753 (72.5%) were sense or antisense oligos designed for transcripts with unknown orientation for which the second probe (antisense or sense respectively) showed good performance.

Cluster analyses were performed on the entire dataset using the AutoSOME strategy [82] by modifying default settings to increase Ensemble runs to 500 and to maintain the p-value threshold at 0.05. A fuzzy cluster network for illustrating the AutoSOME results was generated with the visualisation tool Cytoscape [83]. Bidirectional Hierarchical Clustering (HCL) and Principal Component Analysis (PCA), as implemented in TIGR MultiExperiment Viewer (MeV, version 4.5.1), were also performed on the entire gene expression dataset. Expression profile comparisons between developmental stages were performed using Significance Analysis of Microarrays (SAM) software [15]. Two-class comparisons (FDR 1%, minimal Fold-Change (FC) ≥ 2) were performed by considering each time point as independent. SAM quantitative correlation analyses (FDR 0%) were also performed in order to reveal genes whose expression was positively or negatively correlated with either developmental stages or sample projection on the PCA Y-axis. A non-parametric Spearman rank-correlation test was used to assess the correlation between the expression values measured by real-time RT-PCR and microarray for a set of 10 candidate genes. Spearman correlation tests were implemented using SPSS 12.0.

### Functional annotation of differentially expressed genes

Functional annotation analysis of differentially expressed genes was performed using the DAVID (Database for *Annotation*, Visualisation and Integrated Discovery) web-server [84]. “Biological process”, “Molecular function” and “Cellular component” annotations were performed by setting gene count = 4 and ease = 0.05. KEGG pathway analysis was also performed with gene count = 4 and ease = 0.05. Because DAVID contains functional annotation data for a limited number of species, it was necessary to link sole transcripts with sequence identifiers that could be recognised in DAVID. This was performed using *S. solea* matches with zebrafish proteins and transcripts (see “Transcriptome annotation” section). Finally, *D. rerio* Ensembl Gene IDs were obtained from the corresponding Ensembl protein and transcript entries using the BIOMART data mining tool [85].

### Real-time RT-PCR validation

A set of 10 genes was tested by RT-qPCR using the same samples used for the microarray experiments to validate the performance of the microarray platform. Those genes, listed in Table 2, were chosen from among those found to be involved in larval development and that displayed different patterns of expression across stages. A total of 16 samples, 4 pools for each of 1, 6, 13 and 24 dph larvae, were employed. One microgram of total RNA for each sample was reverse-transcribed to cDNA in a total of 20 μl, by using 200 units of Superscript II (Invitrogen™, Carlsbad, California) and 1 μl random hexamers (50 μM). An aliquot (2.5 μl) of diluted (1:100) cDNA template was amplified in a final volume of 10 μl containing 5 μl Platinum SYBR Green qPCR SuperMix-UDG 2× (Invitrogen™) and 0.25 μl each gene-specific primer (10 μM). The amplification protocol consisted of an initial step of 2 min at 50°C and 2 min at 95° followed by 45 cycles of 10 s at 95°C and 30 s at 60°C. All experiments were performed in a *LightCycler*^®^*480* thermocycler (Roche Diagnostics, Mannheim, Germany). To evaluate the efficiency of each assay, standard curves were constructed by amplifying twofold serial dilutions (from 1:40 to 1:1280) of the same cDNA, which was used as a calibrator. For all transcripts, the efficiency of the primer pairs was always within the range 95 - 105%. For each sample, the Cp (Crossing point) was used to determine the relative amount of target gene; each measurement was performed in duplicate and normalised to the reference gene (ribosomal protein S27, RPS27), which was also measured in duplicate. RPS27 (Isotig21747) was chosen as the reference for RT-qPCR assays as it is commonly considered a housekeeping gene and did not exhibit any significant change in microarray data between developmental stages (%CV 7.5%).

### Microsatellite screening

All annotated Isotigs and contigs were used for microsatellite repeat searches using MISA software [86]. A sequence was considered to contain a microsatellite if it possessed any of the following repeated motifs: at least 6 repeated dinucleotides or at least 5 repeated tri-, tetra-, penta- or hexanucleotide motifs.

## Competing interests

The authors declare that they have no competing interests.

## Authors’ contributions

PPG, LB, SC, PM and AB conceived and designed the project. AB and LP conducted fish rearing and sampling. SF carried out transcriptome sequencing and annotation, probe design, microarray experiments and qRT-PCR validation. SF and LB executed statistical analyses and functional annotation. SF, LB, AB and PPG wrote the manuscript. All listed authors edited the manuscript. All authors read and approved the manuscript.

## Supplementary Material

Additional file 1 **Isotigs and their annotation. List of all 16,371 annotated transcripts and their annotation against 18 different transcript/protein databases. **Different excel sheets for each of the 18 databases used for sequences annotation are provided. In each, species affiliation of hit, accession number, length of aligned region (for match with transcriptomes) and E-value are reported. An additional sheet called “global annotation” reports a global view of all match. Click here for file

Additional file 2 **Heatmaps representing the gene expression value in each developmental stages of pathways and genes listed in Table** 1**. **A. Glucose metabolism, B. Muscle development, C. Hedgehog signaling pathway, D. Wnt signaling pathway. Click here for file

Additional file 3 **Functional annotation of differentially expressed genes across larval transitions.** Lists of Biological processes and KEGG pathways found significantly enriched in each stage comparison. For each comparison three different lists are reported: “ALL” indicates enriched BP or KEGG pathways found enriched when considering all significant genes (both up- and down-regulated), “UP” indicates BP or KEGG pathways found enriched within up-regulated genes while “DOWN” indicates BP or KEGG pathways found enriched within up-regulated genes. Click here for file

Additional file 4 **Phylogenetic analysis of “hatching enzymes”. **Phylogenetic tree showing the evolutionary relationships between S. solea sequences (indicated with Isotig name) and all available astacin-like metalloproteases from vertebrate genomes. Methods on how phylogenetic analysis was conducted are also reported. Click here for file

Additional file 5 **List of significant probes identified by SAM quantitative analysis. **Microarray probes significantly correlated to samples projection on PCA Y-axis. Probes highlighted in red are those found positively correlated to samples projection on PCA Y-axis while probes highlighted in green are those found negatively correlated to samples projection on PCA Y-axis. Annotation for each probe is also reported. Click here for file

Additional file 6 **Functional annotation of genes up-regulated on pre-metamorphic larvae. ** Functional annotation, based on both Gene Ontology and KEGG Pathway analyses, of genes significantly up-regulated in pre-metamorphic larvae and positively correlated to PCA Y-axis projection. Click here for file

Additional file 7 **List and annotation of up-regulated genes on pre-metamorphic larvae. **List and annotation of 372 genes found both significantly up-regulated in pre-metamorphic larvae and positively correlated to samples projection on PCA Y-axis. Click here for file

Additional file 8 **List and annotation of down-regulated genes on pre-metamorphic larvae. **List and annotation of 190 genes found both significantly down-regulated in pre-metamorphic larvae and negatively correlated to samples projection PCA Y-axis. Click here for file
