# Supplementary figures and images for: Exploring the larval transcriptome of the common sole (Solea solea L.)
Source: BMC Genomics. 2013 May 10;14:315. doi: 10.1186/1471-2164-14-315 (PMC3659078; doi:10.1186/1471-2164-14-315)

A.

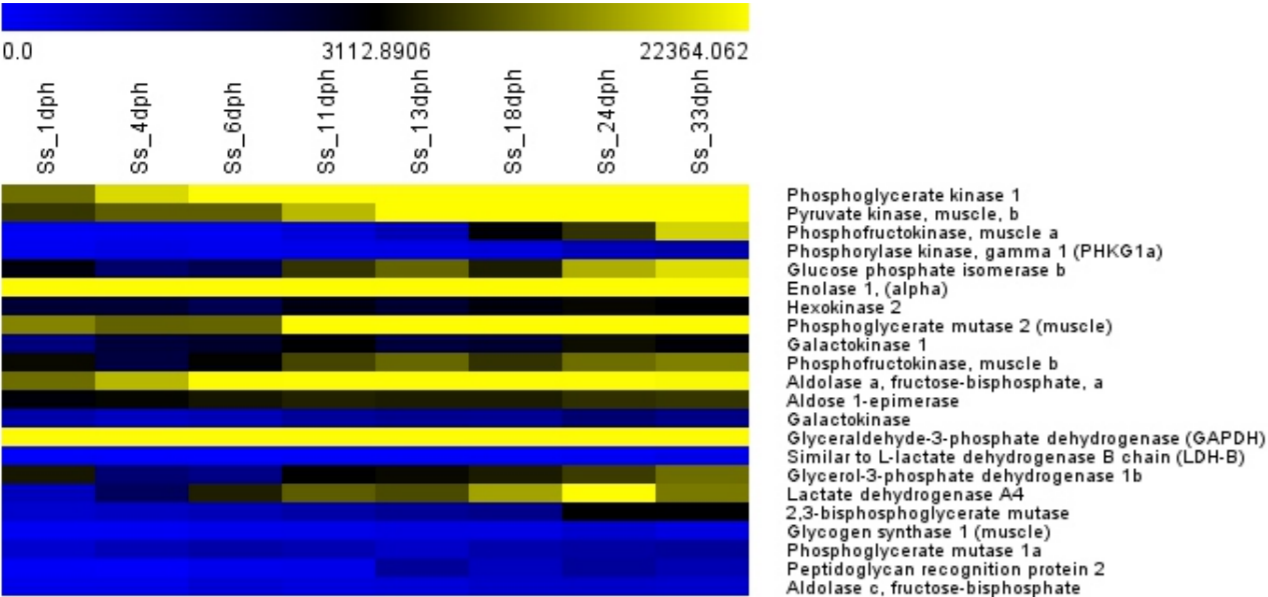

B.

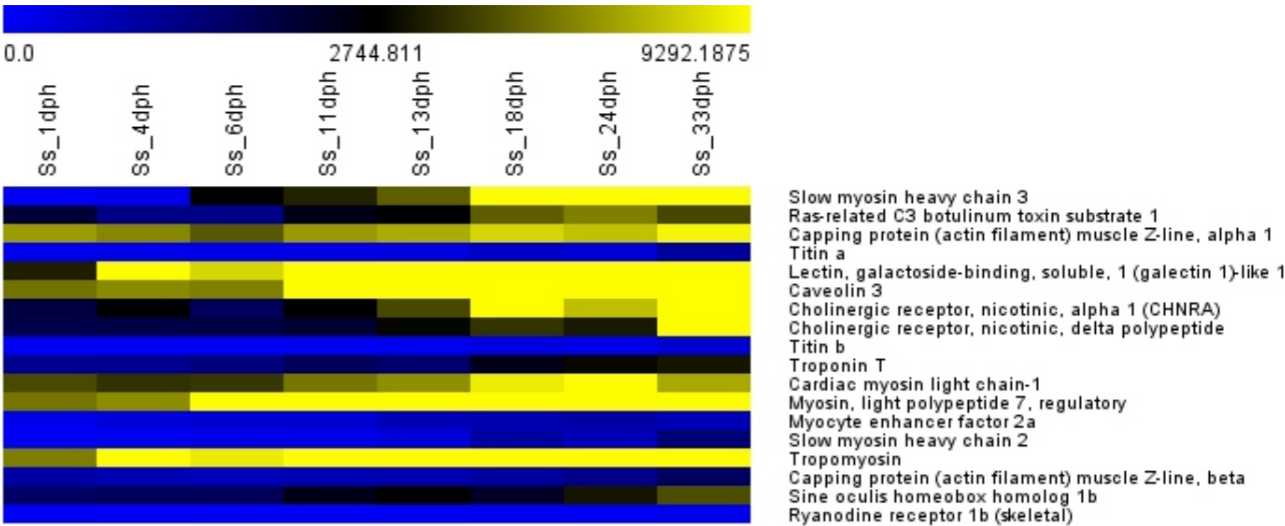

C.

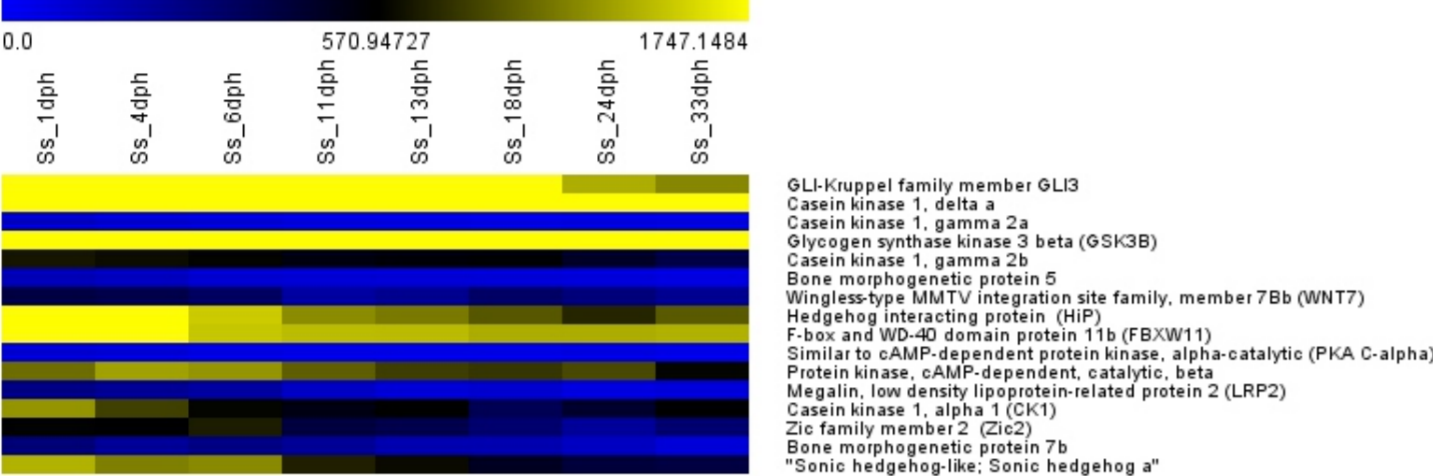

D.

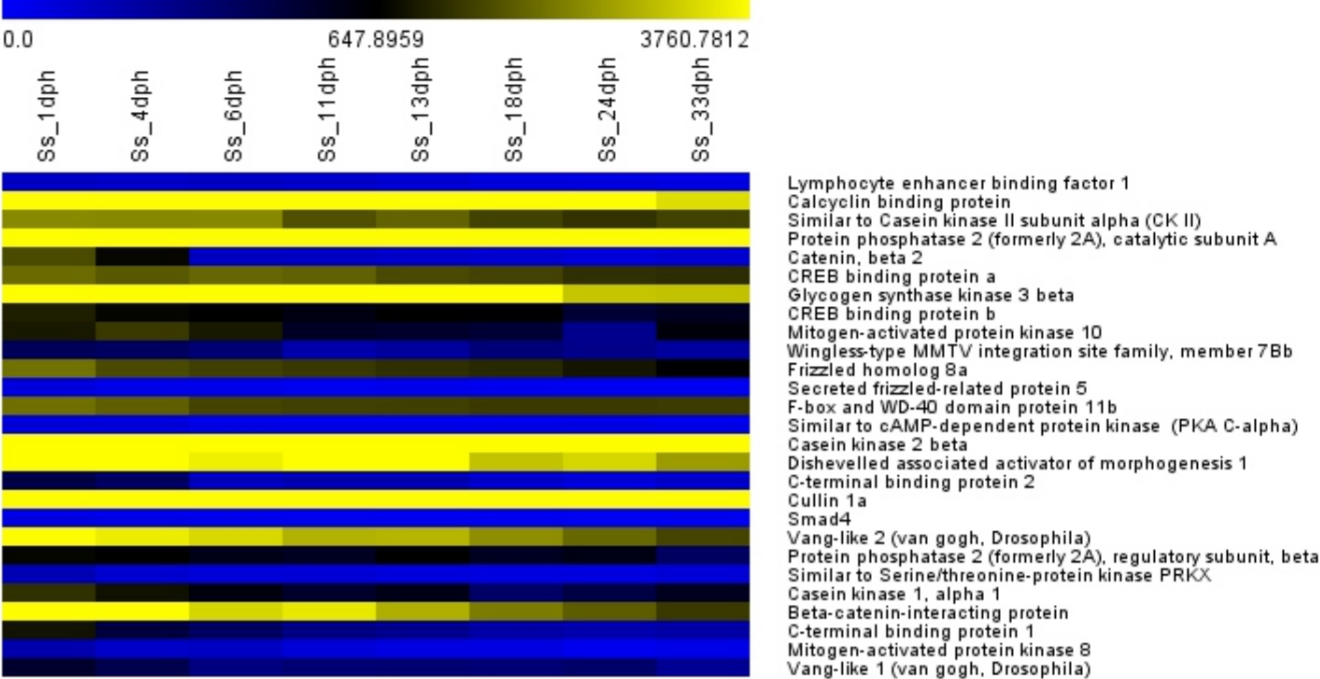

Supplement: Additional file 2 — Heatmaps representing the gene expression value in each developmental stages of pathways and genes listed in Table 1. A. Glucose metabolism, B. Muscle development, C. Hedgehog signaling pathway, D. Wnt signaling pathway. [file 1471-2164-14-315-S2.pdf]
