# Supplementary material for: Exploring the larval transcriptome of the common sole (Solea solea L.)
Source: BMC Genomics. 2013 May 10;14:315. doi: 10.1186/1471-2164-14-315 (PMC3659078; doi:10.1186/1471-2164-14-315)
Supplement: Additional file 4 — Phylogenetic analysis of “hatching enzymes”. Phylogenetic tree showing the evolutionary relationships between S. solea sequences (indicated with Isotig name) and all available astacin-like metalloproteases from vertebrate genomes. Methods on how phylogenetic analysis was conducted are also reported. [file 1471-2164-14-315-S4.pdf]

# Phylogenetic analysis of "hatching" enzymes

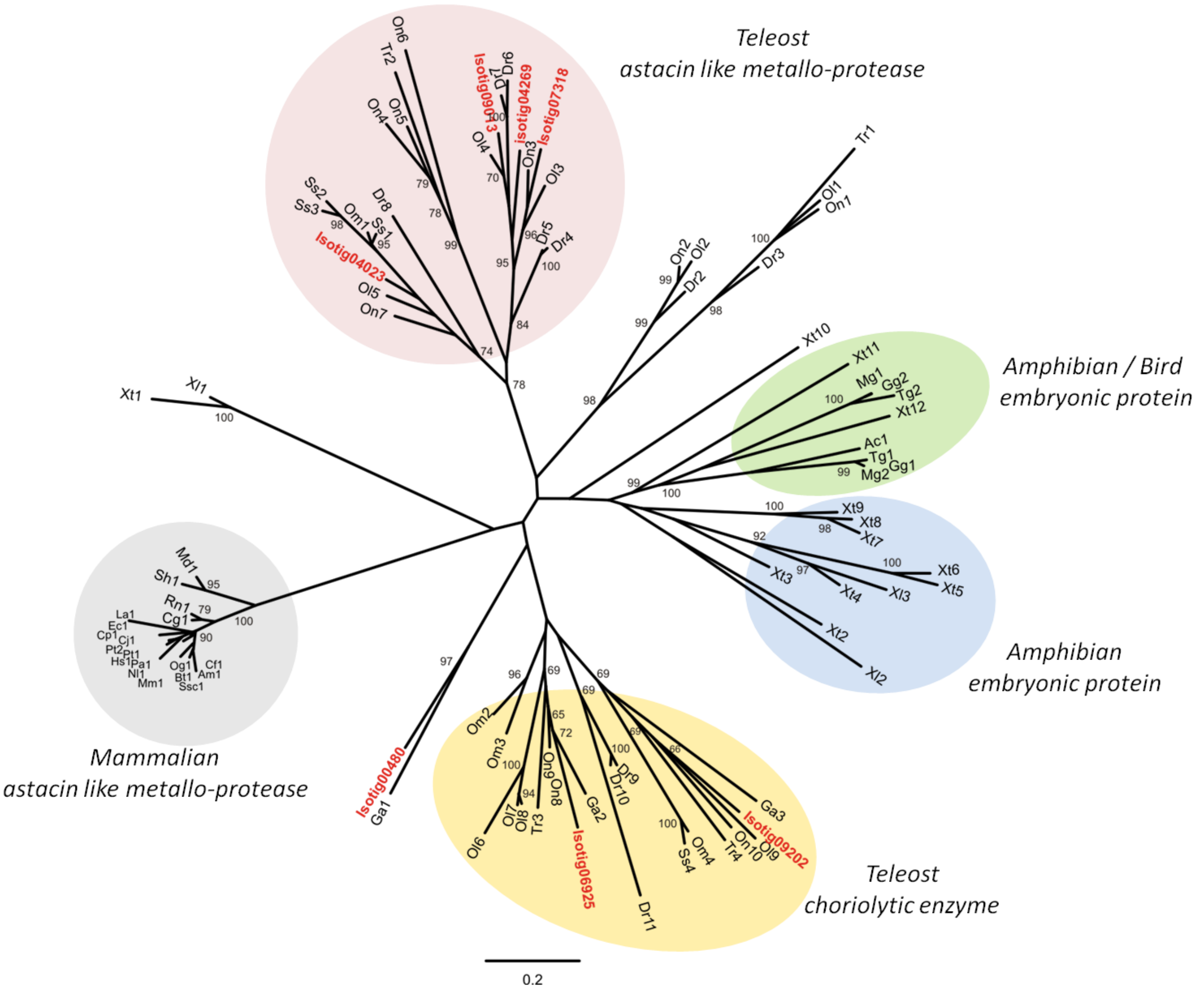


Phylogenetic tree showing the evolutionary relationships between *S. solea* sequences (indicated with Isotig name) and all available astacin-like metalloproteases from vertebrate genomes. Vertebrate sequences are defined by the two-letter species followed by a number, correspondence of this "code" with each gene description and accession number on GenBank is reported on the Table below. Bootstrap values are assigned to each interior branch; bootstrap values less than 60 are not shown.

## Methods

Translated *S. solea* hatching enzymes sequences were used as query for Blastp searches in NCBI nr protein database in order to find putative orthologs in other species. A total of 89 sequences (see below) were then employed for phylogenetic analysis, 4 sequences were later excluded since too divergent and barely aligneable.

S. solea hatching enzymes and all published protein sequences were aligned using MUSCLE (MUltiple Sequence Comparison by Log- Expectation) software, applying default settings. The maximum likelihood analysis was performed with the Phyml 3.0 programme by applying the LG+I+G evolutionary model; non-parametric bootstrap resampling was also performed to evaluate the robustness of tree topology.

*List of sequences employed for phylogenetic tree construction.*

| Code in phylogenetic tree | GenBank Acc. | Description |
|---|---|---|
| **AC1** | XP_003214688.1 | PREDICTED: embryonic protein UVS.2-like [Anolis carolinensis] |
| **AM1** | XP_002926533.1 | astacin-like metalloendopeptidase-like [Ailuropoda melanoleuca] |
| **BT1** | XP_588342.2 | PREDICTED: astacin-like metalloendopeptidase [Bos taurus] |
| **CF1** | XP_003639630.1 | PREDICTED: astacin-like metalloendopeptidase-like [Canis lupus familiaris] |
| **CG1** | XP_003497858.1 | PREDICTED: astacin-like metalloendopeptidase [Cricetulus griseus] |
| **CJ1** | XP_002757421.1 | PREDICTED: astacin-like metalloendopeptidase [Callithrix jacchus] |
| **CP1** | XP_003471753.1 | PREDICTED: astacin-like metalloendopeptidase-like [Cavia porcellus] |
| **DR10** | NP_001038639.1 | hatching enzyme 1a precursor [Danio rerio] |
| **DR11** | NP_001091658.1 | hatching enzyme 2 precursor [Danio rerio] |
| **DR2** | XP_001919316.2 | PREDICTED: high choriolytic enzyme 1-like [Danio rerio] |
| **DR3** | XP_001342763.2 | PREDICTED: high choriolytic enzyme 1-like [Danio rerio] |
| **DR4** | NP_991145.1 | nephrosin-like precursor [Danio rerio] |
| **DR5** | NP_001071247.1 | nephrosin isoform 1 precursor [Danio rerio] |
| **DR6** | NP_001013544.1 | six-cysteine containing astacin protease 3 precursor [Danio rerio] |
| **DR7** | NP_001020351.1 | six-cysteine containing astacin protease 4 precursor [Danio rerio] |
| **DR8** | NP_001036784.1 | six-cysteine containing astacin protease 1 precursor [Danio rerio] |
| **DR9** | NP_998800.2 | hatching enzyme 1b precursor [Danio rerio] |
| **EC1** | XP_001916411.2 | astacin-like metalloendopeptidase [Equus caballus] |
| **GA1** | ENSGACP00000004002 (Ensembl acc. Number) | |
| **GA2** | NP_001254602.1 | hatching enzyme precursor [Gasterosteus aculeatus] |
| **GA3** | NP_001254603.1 | hatching enzyme precursor [Gasterosteus aculeatus] |
| **GG1** | XP_426424.3 | PREDICTED: embryonic protein UVS.2-like [Gallus gallus] |
| **GG2** | XP_421101.2 | PREDICTED: astacin-like metalloendopeptidase [Gallus gallus] |
| **HS1** | NP_001002036.3 | astacin-like metalloendopeptidase precursor [Homo sapiens] |
| **LA1** | XP_003422064.1 | PREDICTED: astacin-like metalloendopeptidase-like [Loxodonta africana] |
| **MD1** | XP_001382060.1 | PREDICTED: astacin-like metalloendopeptidase-like [Monodelphis domestica] |
| **MG1** | XP_003206449.1 | PREDICTED: astacin-like metalloendopeptidase-like [Meleagris gallopavo] |
| **MG2** | XP_003206490.1 | PREDICTED: embryonic protein UVS.2-like [Meleagris gallopavo] |
| **MM1** | XP_002799475.1 | PREDICTED: astacin-like metalloendopeptidase-like [Macaca mulatta] |
| **NL1** | XP_003281094.1 | PREDICTED: astacin-like metalloendopeptidase [Nomascus leucogenys] |
| **OG1** | XP_003799356.1 | PREDICTED: astacin-like metalloendopeptidase [Otolemur garnettii] |
| **OL1** | NP_001098208.1 | astacin like metallo-protease precursor [Oryzias latipes] |
| **OL2** | NP_001098332.1 | astacin like metallo-protease precursor [Oryzias latipes] |
| **OL3** | NP_001098331.1 | astacin like metallo-protease precursor [Oryzias latipes] |
| **OL4** | NP_001098207.1 | astacin like metallo-protease precursor [Oryzias latipes] |
| **OL5** | NP_001098206.1 | nephrosin precursor [Oryzias latipes] |
| **OL6** | NP_001098205.1 | hatching enzyme like protease precursor [Oryzias latipes] |
| **OL7** | NP_001098293.1 | high choriolytic enzyme 2 precursor [Oryzias latipes] |
| **OL8** | NP_001188427.1 | high choriolytic enzyme 1 precursor [Oryzias latipes] |
| **OL9** | NP_001098292.1 | low choriolytic enzyme precursor [Oryzias latipes] |
| **OM1** | NP_001158583.1 | High choriolytic enzyme 1 precursor [Oncorhynchus mykiss] |
| **OM2** | NP_001165879.1 | hatching enzyme 2 precursor [Oncorhynchus mykiss] |

| | | |
|---|---|---|
| **OM3** | NP_001165880.1 | hatching enzyme 1 precursor [Oncorhynchus mykiss] |
| **OM4** | NP_001165878.1 | hatching enzyme precursor [Oncorhynchus mykiss] |
| **ON1** | XP_003447393.1 | PREDICTED: low choriolytic enzyme-like [Oreochromis niloticus] |
| **ON10** | XP_003440698.1 | PREDICTED: low choriolytic enzyme-like [Oreochromis niloticus] |
| **ON2** | XP_003447395.1 | PREDICTED: high choriolytic enzyme 2-like [Oreochromis niloticus] |
| **ON3** | XP_003449256.1 | PREDICTED: hypothetical protein LOC100697799 [Oreochromis niloticus] |
| **ON4** | XP_003459646.1 | PREDICTED: high choriolytic enzyme 1-like, partial [Oreochromis niloticus] |
| **ON5** | XP_003456934.1 | PREDICTED: high choriolytic enzyme 1-like [Oreochromis niloticus] |
| **ON6** | XP_003456946.1 | PREDICTED: low choriolytic enzyme-like [Oreochromis niloticus] |
| **ON7** | XP_003458522.1 | PREDICTED: low choriolytic enzyme-like [Oreochromis niloticus] |
| **ON8** | XP_003440757.1 | PREDICTED: high choriolytic enzyme 2-like [Oreochromis niloticus] |
| **ON9** | XP_003440875.1 | PREDICTED: high choriolytic enzyme 1-like [Oreochromis niloticus] |
| **PA1** | XP_002811693.1 | PREDICTED: astacin-like metalloendopeptidase [Pongo abelii] |
| **PT1** | XP_003309175.1 | astacin-like metalloendopeptidase isoform 2 [Pan troglodytes] |
| **PT2** | XP_001144288.1 | astacin-like metalloendopeptidase isoform 1 [Pan troglodytes] |
| **RN1** | NP_001099974.1 | astacin-like metalloendopeptidase precursor [Rattus norvegicus] |
| **SH1** | XP_003758159.1 | PREDICTED: uncharacterized protein LOC100929627 [Sarcophilus harrisii] |
| **SS1** | NP_001134276.1 | High choriolytic enzyme 1 precursor [Salmo salar] |
| **SS2** | NP_001140025.1 | High choriolytic enzyme 2 precursor [Salmo salar] |
| **SS3** | NP_001158733.1 | Low choriolytic enzyme precursor [Salmo salar] |
| **SS4** | NP_001156553.1 | hatching enzyme precursor [Salmo salar] |
| **SSc1** | XP_003481181.1 | PREDICTED: astacin-like metalloendopeptidase-like [Sus scrofa] |
| **TG1** | XP_002198927 | embryonic protein UVS.2-like [Taeniopygia guttata] |
| **TG2** | XP_002198668 | astacin-like metalloendopeptidase-like [Taeniopygia guttata |
| **TR1** | NP_001072095.1 | astacin like metallo-protease precursor [Takifugu rubripes] |
| **TR2** | NP_001072094.1 | astacin like metallo-protease precursor [Takifugu rubripes] |
| **TR3** | NP_001072106.1 | hatching enzyme precursor [Takifugu rubripes] |
| **TR4** | NP_001072065.1 | hatching enzyme precursor [Takifugu rubripes] |
| **XL1** | NP_001108257.1 | uncharacterized protein LOC100137635 precursor [Xenopus laevis] |
| **XL2** | NP_001121238.1 | uncharacterized protein LOC100158314 [Xenopus laevis] |
| **XL3** | NP_001088112.1 | uncharacterized protein LOC494813 precursor [Xenopus laevis] |
| **XT1** | XP_002932488.1 | PREDICTED: bone morphogenetic protein 1-like [Xenopus tropicalis] |
| **XT10** | XP_002938079.1 | PREDICTED: embryonic protein UVS.2-like [Xenopus tropicalis] |
| **XT11** | XP_002937346.1 | PREDICTED: embryonic protein UVS.2-like [Xenopus tropicalis] |
| **XT12** | XP_002934133.1 | PREDICTED: embryonic protein UVS.2-like [Xenopus tropicalis] |
| **XT2** | XP_002937348.1 | PREDICTED: embryonic protein UVS.2-like [Xenopus tropicalis] |
| **XT3** | XP_002934115.1 | PREDICTED: embryonic protein UVS.2-like [Xenopus tropicalis] |
| **XT4** | NP_001008039.1 | tolloid-like 1 precursor [Xenopus tropicalis] |
| **XT5** | XP_002934134.1 | PREDICTED: embryonic protein UVS.2-like [Xenopus tropicalis] |
| **XT6** | XP_002934137.1 | PREDICTED: embryonic protein UVS.2-like [Xenopus tropicalis] |
| **XT7** | XP_002937467.1 | tolloid-like protein 2-like [Xenopus tropicalis] |
| **XT8** | XP_002937432.1 | PREDICTED: embryonic protein UVS.2-like [Xenopus tropicalis] |
| **XT9** | XP_002937431.1 | PREDICTED: embryonic protein UVS.2-like [Xenopus tropicalis] |
